# Complex soliton wave patterns of Gross–Pitaevskii systems: application in quantum and optical engineering

**DOI:** 10.1038/s41598-025-27902-0

**Published:** 2025-12-22

**Authors:** Muhammad Bilal, Yazen M. Alawaideh, Shafqat Ur Rehman, Ioan-Lucian Popa, Bashar M. Al-khamiseh, Hala Ghannam

**Affiliations:** 1 Department of Physics, Shanghai University, Shanghai, 200444 China; 2 Applied Science Research Center, Applied Science Private University, Amman, 11931 Jordan; 3 Department of Mathematics, Grand Asian University, Sialkot, 51310 Pakistan; 4 Department of Computing, Mathematics and Electronics, 1 Decembrie 1918 University of Alba Iulia, 510009 Alba Iulia, Romania; 5 Faculty of Mathematics and Computer Science, Transilvania University of Brasov, Iuliu Maniu Street 50, 500091 Brasov, Romania; 6 Department of Basic Sciences, Middle East University, Amman, 11831 Jordan; 7 Department of Physics, The University of Jordan, Amman, Jordan

**Keywords:** Solitary wave solutions, Multicomponent Gross–Pitaevskii system, New extended hyperbolic function method, Condensed matter physics, $\beta$-derivative, Mathematics and computing, Physics

## Abstract

The purpose of this work is to explore precise solutions, particularly soliton solutions, by fractionally analyzing the multicomponent Gross–Pitaevskii problem, a basic nonlinear Schrödinger equation. Soliton solutions are essential for comprehending the complex system dynamics, providing insight into superfluidity, superconductivity, and related nonlinear effects. The complex fractional Gross–Pitaevskii equation is solved by considering the $\beta$-derivative. Numerous optical solutions, including trigonometric, hyperbolic, rational function, and complex multiple soliton structures, are achieved by using elegant integration methods. All reported solutions are verified using the Wolfram Mathematica program by putting back-substitution into the governing equation. The novel solutions are acquired, capitalizing on the new extended hyperbolic function method (EHFM) and the unified method that holds significant implications across various scientific disciplines. Furthermore, we portray various wave profiles with the corresponding parametric values under the influence of $\beta$-derivative. This visualization style enhances our understanding of the acquired solutions and facilitates a thorough examination of their potential practical applications. By engaging the aforementioned cutting-edge methods, we obtain an effective framework for analyzing the complex nonlinear phenomena that emerge in several physical contexts.

## Introduction

Ultracold interactions have been extensively studied in many different quantum fluids such as ultracold Fermi gases, superfluid 3He and 4He, and Bose–Einstein condensates (BECs) in the last two decades of ultracold quantum physics research^1,2^. In these systems, rich nonlinear dynamics emerge from the interplay between the interacting particles, and these interactions are modulated by the external trapping potential. Recently, the realization of Feshbach resonance techniques that can provide precise control of interactions in ultracold Fermi gases^3^, which can be continuously tuned from the Bardeen-Cooper-Schrieffer (BCS) state of weakly paired fermions to the molecular BEC regime^4^, has greatly enhanced the utility of ultracold quantum gases as a “desktop laboratory” for the study of prototype phenomena in many areas of great interest ranging from many-body quantum systems to condensed matter physics (CMP), superconductivity and superfluidity, as well as providing motivations for studies of analogous phenomena in astrophysical systems.

As a comprehensive model in mean-field theory Gross–Pitaevskii equation (GPE) has been extensively studied to reveal the BEC properties at ultracold temperature, and exact solutions showed rich soliton patterns including bright and dark soliton patterns^5,6^. The GPE has proved its usefulness for the understanding of the BEC dynamics in cold atomic Fermi gases, but a thorough study of the soliton solutions of GPE is still wanting^7,8^. In view of the above motivations, this study attempts to improve our understanding of the BEC dynamics and its applications in CMP by generating and studying the GPE exact wave solutions in a systematic way. It is well known that the GPE is an important model linking nonlinear optics, nonlinear science and wave physics. The GPE can explain the necessary phenomena such as four wave mixing and soliton phenomena in BEC. Therefore, it is very important to study the GPE in details to understand the precise predictions and applications in quantum physics research^9,10^.

Nonlinear partial differential equations (NLPDEs) perform as influential mathematical tools for modeling intricate phenomena across various scientific and engineering realms, including astronomy, biological systems, chemical processes, quantum mechanics, fiber optics, and fluid dynamics^11–16^. These equations offer rigorous frameworks for evaluating nonlinear physical systems, enabling exact predictions of wave propagation shapes and system behaviors^17–21^. Researchers have established abundant analytical approaches to solve NLPDEs, including the Hirota bilinear technique, $\exp$-function approach, $G'/G$-expansion method, solitary wave ansatz, generalized Riccati equation mapping scheme, $\tan -\cot$ approach, Weierstrass elliptic functions, improved $\tan \frac{\phi ({\eta })}{2}$ method, Lie symmetry analysis, and Darboux transformations^22–29^. These solution techniques have demonstrated particularly valuable in nonlinear optics, where soliton theory plays a key role in advancing telecommunications technologies, metasurfaces, magneto-optic waveguide systems, and metamaterials. Moreover, some researchers have explored nonlinear problems through analytical, numerical, and data-driven approaches via Dbar-dressing and asymptotic methods^30,31^, neural network approximations^32^, and symbolic, genetic-algorithm-based PDE discovery^33^.

The propagation of optical solitons through fiber-optic cables over transcontinental distances signifies a significant area of modern photonics research, governed by complex NLPDEs that define their unique wave dynamics^34,35^. These self-sustaining wave packets sustain their shape through a precise balance between dispersion and nonlinear effects, enabling high-capacity data transmission crucial for global telecommunications infrastructure. With the help of the optical fiber’s two-layer construction-made up of a core that carries light and cladding that reflects it-total internal reflection allows soliton pulses to be guided with minimal signal degradation, while the protective polyvinyl coatings confirm durability. Outside of telecommunications, major potential areas of application for soliton-based systems are medical imaging, industrial inspection, and automotive lighting-all domains that are more concerned with operation without thermal effects and flexibility. The current research focuses mainly on developing new soliton natures with improved stability properties-especially for applications in birefringent fibers, magneto-optical devices, and metamaterials-where exact analytical solutions continue to be of great value in correlating theoretical estimates with experimental observations of nonlinear optical systems.

In order to bridge this gap in the literature, in this study we apply the new EHFM^36^ and the unified technique^37^ for finding novel soliton solutions of the considered model. The EHFM and the unified method are, respectively, new analytical techniques for the construction of exact solutions to NLPDEs. The new EHFM is concerned with the solutions in terms of hyperbolic and usually trigonometric functions by the assumption of a certain ansatz. On the other hand, the unified method offers a more general approach to derive in a straightforward manner a broader class of solutions such as solitary, periodic, and rational forms by a single algorithmic prototype. Thus, the two methods increase the diversity and the robustness of the solutions in nonlinear modeling. These two new techniques have the advantage over the conventional methods. The new techniques are free from the defects of the conventional approaches and yield new complete solutions. Some of the advantages are obtaining a simplified algorithm for various explicit and precise solutions, improved efficiency when handling complicated nonlinear models, and reducing the computational complexity. These advantages are significant and yield considerable outputs to us in understanding real situations and appreciate more. It is imperative to verify the effectiveness of the newly proposed EHFM and the Unified method in obtaining the soliton solutions. The instability analysis pinpoints the regime in which the obtained solutions are physically stable and dynamically robust under small perturbations. That is, it is required to verify whether the soliton profiles remain stable over time and do not change their shapes during propagation by either performing the linear stability analysis or the numerical time evolution simulations. This would establish the range of accuracy and reliability of the two analytical methods and also the range of applicability of the two methods in describing realistic nonlinear wave phenomena. Hence, establishing the stability of the stable solitons is a vital step in the success range of both EHFM and the Unified method. Our original research provides the way for future studies in a variety of nonlinear media by offering a wide range of accessible analytical solutions and groundbreaking methods for generating optical wave characteristics.

The sequence of the remaining article is arranged as: Sect. 2 discusses conformable derivatives and their properties. Section 3 establishes the studied model, whereas Sect. 4 describes the mathematical structure and application of the unique EHFM and unified technique. Section 5 includes graphical representations of achieved solutions. The role of complex soliton structures in physical systems is given in Sect. 6. Finally, Sect. 7 provides an overview of major findings and implications for future research.

## Fractional order derivative

The analysis of complex natural systems from wave propagation in diverse media to spatio-temporal population dynamics fundamentally depends on PDEs. With modern technological systems growing in sophistication, traditional integer-order PDEs often prove insufficient to capture multiscale memory effects and nonlocal interactions. This limitation has driven the adoption of fractional-order PDEs, enabled by advanced fractional operators like the $\beta$-derivative, which inherently encode system history and cross-scale coupling through their noninteger differentiation orders. Current studies highlight the superiority of $\beta$ fractional derivative over other fractional derivatives in representing the dynamics of solitary waves in nonlinear systems. These tailored derivatives provide a powerful tool for intricate occurrences in a variety of biological, physical, and engineering contexts. To provide precise solutions for the model under consideration, this study makes use of such a derivative. This allows for a comparative examination of various solution types and provides insight into their dynamic characteristics.

**The** $$\beta$$ **-derivative**

### Definition 2.1

For $x(t): [c,\infty )\rightarrow \mathbb {R}$, the $\beta$-derivative^38^ of *x* is described by:

1 $$\begin{aligned} \mathscr {D}_{t}^{\varrho }\{x(t)\}=\lim _{\epsilon \rightarrow 0}\frac{x\left( t+\epsilon \left( t+\frac{1}{\Gamma (\varrho )}\right) ^{1-\varrho }\right) -x(t)}{\epsilon },~~~0<\varrho \le 1. \end{aligned}$$

### Theorem 2.2

^38^ Assume that $y\ne 0$ and *x* are two differentiable functions with $0<\varrho \le 1$, such that:

1. $$\mathscr {D}_{t}^{\varrho }\left( c\{x(t)\}+d\{ y(t)\}\right) =c\mathscr {D}_{t}^{\varrho }\{x(t)\}+d\mathscr {D}_{t}^{\varrho }\{y(t)\},~\forall c,~d\in \mathbb {R}$$.
2. $$\mathscr {D}_{t}^{\varrho }\left( \{y(t)\}\times \{x(t)\} \right) = \{y(t)\}\mathscr {D}_{t}^{\varrho }\left( \{x(t)\}\right) +\{x(t)\}\mathscr {D}_{t}^{\varrho }\left( \{y(t)\}\right)$$.
3. $$\mathscr {D}_{t}^{\varrho }\left( \frac{\{x(t)\}}{\{y(t)\}}\right) =\frac{\{y(t)\}\mathscr {D}_{t}^{\varrho }\{x(t)\}-\{x(t)\}\mathscr {D}_{t}^{\varrho }\{y(t)\}}{\left( \{y(t)\}\right) ^2}$$.
4. $$\mathscr {D}_{t}^{\varrho }\{x(t)\}=\frac{d\{x(t)\}}{dt}\left( t+\frac{1}{\Gamma (\varrho )}\right) ^{1-\varrho }$$.
5. $$\mathscr {D}_{t}^{\varrho }\{c(t)\}=0, ~~{\text {with }c \text {is a constant function}}.$$

The consideration of the $\beta$-derivative with respect to standard fractional operators such as Caputo or Riemann-Liouville derivatives implies a kind of compromise; analytical tractability is emphasized while still retaining physical relevance. While Caputo and Riemann-Liouville operators are good at modeling memory effects due to their non-local integral structures, the mathematical intricacy of these operators often inhibits finding closed-form solutions and obscures physical interpretation. In contrast, the $\beta$-derivative maintains local differential operations and obeys the conventional rules of calculus, which enables the effective computation of exact solutions while modeling an essential class of fractional-order dynamics. This justification proves particularly advantageous in the study of optical soliton propagation in the three-component GP equations where the values of reduced analysis and an interpretable physical explanation outweigh the need for explicit representation of memory effects. In general, the $\beta$-derivative maintains a balance of mathematical simplicity and modeling capabilities that renders it ideally suitable for nonlinear wave phenomena where the existence of solutions, stability analysis, and parameter dependencies become major issues. The limitations on the hypothesis and the constraints on applicability have been highlighted for consistency and well-posedness of the operator in the wider context of fractional calculus.

## The governing model

Recent developments in ultracold quantum physics beyond the standard single-component BECs distinctly highlighted the two-component BECs, namely spinor condensates, as a rapidly developing area in the investigation of nonlinear wave phenomena. These quantum systems, formed by dilute atomic gases trapped in nanokelvin temperatures, display amazing macroscopic quantum behaviors by means of superfluidity and phase coherence. Recently it has been found out that the spinor BEC contains large internal degrees of freedom and may form complicated quantum states with phase coherence over macroscopic scales. In addition, in the typical one-dimensional optical trap systems, the structure of macroscopic quantum states of such systems appears to be two fundamental stationary states: (1) ferromagnetic states, which are described by one-component wavefunctions with all spins aligned, and (2) polar states, which are viewed as three-component superpositions with certain spin configurations. The relative stability and dynamics of these quantum phases are governed by the relative strengths of s-wave scattering lengths in different angular momentum channels, which control the character of interatomic collisions and further affect the macroscopic quantum behaviors of the condensate.

The three-component (tc) GP system represents a significant generalization of the conventional single- and two-component GP equations, which describe the macroscopic wave functions of BECs. Its originality lies in capturing multi-mode, spinor, or multi-species condensate dynamics where three coupled order parameters interact through nonlinear self- and cross-phase modulation terms. The tc-GP system^39,40^ with the $\beta$-deivative reads

2 $$\begin{aligned} {\left\{ \begin{array}{ll} i D^{\varrho }_{ t} \Upsilon _{+1}+ D^{2\varrho }_{x} \Upsilon _{+1}-2(|\Upsilon _{+1}|^{2}+2|\Upsilon _{0}|^{2})\Upsilon _{+1} -2 \Upsilon ^*_{-1}\Upsilon ^2_{0} =0.\\ i D^{ \varrho }_{ t} \Upsilon _{0}+D^{2\varrho }_{ x} \Upsilon _{0}-2(|\Upsilon _{+1}|^{2}+|\Upsilon _{-1}|^{2}+|\Upsilon _{0}|^{2})\Upsilon _{0}-2\Upsilon ^*_{0}\Upsilon _{-1}\Upsilon _{+1}=0.~~~~~\\ i D^{\varrho }_{ t} \Upsilon _{-1}+ D^{2\varrho }_{x} \Upsilon _{-1}-2(|\Upsilon _{-1}|^{2}+2|\Upsilon _{0}|^{2})\Upsilon _{-1}-2 \Upsilon ^*_{+1}\Upsilon ^2_{0}=0. \end{array}\right. } \end{aligned}$$

The wave functions $\Upsilon _{j}(x, t)$ describe atoms with magnetic spin quantum numbers $j=+1, 0, -1$, where subscripts *x* and *t* denote partial derivatives and * represents the complex conjugate. The notation $D^{\varrho }$ signifies $\beta$-derivative, with $\varrho >0$. New investigations have studied multicomponent BEC systems through multiple analytical gadgets, especially advancing our understanding of nonlinear matter waves. Dark soliton solutions have been systematically derived using Lax pair formulations and Darboux transformation scheme^41^, while nonautonomous vector soliton dynamics have been characterized through exact one- and two-soliton solutions^42^. The inverse scattering transform and Hirota bilinear methods have offered powerful contexts for analyzing these quantum systems^43^, complemented by the extended sinh-Gordon equation expansion approach^44^ and direct algebraic mechanism^45^. Recently, the study in^46^ employed two analytical techniques, the modified Sardar sub-equation method and the enhanced modified extended tanh-expansion method, to extract dispersive wave solutions. Building upon these progresses, the present work extends the theoretical framework by examining the $\beta$-fractional formulation of the three-component GP equations, empowering the derivation of novel solitary wave solutions that widen our understanding of quantum nonlinear phenomena in spinor condensates.

## Extraction of soliton solutions

For obtaining the solutionsof the given system, with the hypothesis:

3 $$\begin{aligned} {\left\{ \begin{array}{ll} \Upsilon _{+1}(x, t)=p(\eta )e^{ i\psi }. \\ \Upsilon _{0}(x. t)=q(\eta )e^{ i\psi }.\\ \Upsilon _{-1}(x, t)=r(\eta )e^{ i\psi }, \end{array}\right. } \end{aligned}$$

where

$$\begin{aligned} \eta =\frac{1 }{\varrho }\left( x+\frac{1}{\Gamma (\varrho )}\right) ^{\varrho }-\frac{\varsigma }{\varrho }\left( t+\frac{1}{\Gamma (\varrho )}\right) ^{\varrho }~~\text{ and }~~ \psi = \frac{\sigma }{\varrho }\left( x+\frac{1}{\Gamma (\varrho )}\right) ^{\varrho }+\frac{\omega }{\varrho }\left( t+\frac{1}{\Gamma (\varrho )}\right) ^{\varrho }. \end{aligned}$$

Here, the real constants are $\varrho$, $\sigma$ and $\omega$. By taking the defined transformation for Eqs. (2), we attain the real part

4 $$\begin{aligned} {\left\{ \begin{array}{ll} 2 p^3+p \left( \sigma ^2+4 q^2+\omega \right) +2 q^2 r- p''=0.\\ ~-2 q^3-q \left( \sigma ^2+2 \left( p^2+ p r+r^2\right) +\omega \right) +q''=0.\\ ~ 2 r^3+2 p q^2+r \left( \sigma ^2+4 q^2 +\omega \right) -r''=0. \end{array}\right. } \end{aligned}$$

From imaginary part we get $\varsigma =2 \sigma$. Applying the balance principle formula on $p^3, q^3, r^3$ and $p'', q'', r''$ in Eq. (4) yields, $n=1$.

### New EHFM

The various segments of the new EHFM are outlined below:

- *Step 1:* Suppose a NLPDE
  5 $$\begin{aligned} R(S, S_{t}, S_{x}, S_{tt}, S_{xt}, S_{xx},...)=0. \end{aligned}$$
  Where *R* denotes a polynomial of *S*(*x*, *t*) with its inputs.
- *Step 2:* If we consider
  6 $$\begin{aligned} S(x,t)=G(\eta )e^{ i\psi }. \end{aligned}$$
  Where
  $$\begin{aligned} \eta =\frac{1 }{\varrho }\left( x+\frac{1}{\Gamma (\varrho )}\right) ^{\varrho }-\frac{\varsigma }{\varrho }\left( t+\frac{1}{\Gamma (\varrho )}\right) ^{\varrho }~~\text{ and }~~ \psi = \frac{\sigma }{\varrho }\left( x+\frac{1}{\Gamma (\varrho )}\right) ^{\varrho }+\frac{\omega }{\varrho }\left( t+\frac{1}{\Gamma (\varrho )}\right) ^{\varrho }. \end{aligned}$$
  The Eq. (5) reduces an ordinary differentail equation (ODE) under the Eq. (6).
  7 $$\begin{aligned} \Phi (G,G', G'', G''', \cdots )=0. \end{aligned}$$
  Where $\Phi$ indicates a polynomial function of *G* and its derivatives. **Form 1:**
- *Step 3:* Let the solution to Eq. (7), in the following form:
  8 $$\begin{aligned} G(\eta )=\sum _{k=0}^n F_k Q(\eta )^k. \end{aligned}$$
  Where $F_k(k = 0, 1, 2, 3, \cdots n )$ are the constants and $Q(\eta )$ admits the ODE to be written as:
  9 $$\begin{aligned} \frac{dQ}{d\eta } =Q \sqrt{\tau +\mu Q^2} ~~~\tau ,\mu \in \Re . \end{aligned}$$
  Applying the balance principle formula to Eq. (7) for finding the value of *n*. Inserting Eq. (8) into Eq. (7), we get a set of nonlinear equations for $F_k$ ($k = 0, 1, 2, 3, \ldots , n$), with the solutions sets that permits Eq. (9).
  10 $$\begin{aligned} & {\textbf {Set 1:}}~\text {For}~~ \tau>0 ~~\text {and} ~~\mu >0,~~\text {we have}~~Q(\eta ) =- \sqrt{\frac{\tau }{\mu }}\text {csch} (\sqrt{\tau } (\eta +k_0 ). \end{aligned}$$
  11 $$\begin{aligned} & {\textbf {Set 2:}}~\text {For}~~ \tau <0 ~~\text {and}~~ \mu >0,~~\text {we have}~~ Q(\eta ) = \sqrt{\frac{-\tau }{\mu }}\text {sec} (\sqrt{-\tau } (\eta +k_0). \end{aligned}$$
  12 $$\begin{aligned} & {\textbf {Set 3:}}~\text {For}~~ \tau >0 ~~\text {and}~~ \mu <0,~~\text {we have}~~ Q(\eta ) = \sqrt{\frac{\tau }{- \mu }}\text {sech} (\sqrt{\tau } (\eta +k_0). \end{aligned}$$
  13 $$\begin{aligned} & {\textbf {Set 4:}}~\text {For}~~ \tau <0 ~~\text {and} ~~\mu >0,~~\text {we have}~~ Q(\eta ) = \sqrt{\frac{-\tau }{ \mu }}\text {csc} (\sqrt{-\tau } (\eta +k_0 ). \end{aligned}$$
  14 $$\begin{aligned} & {\textbf {Set 5:}}~\text {For}~~ \tau >0 ~~\text {and}~~\mu =0,~~\text {we have}~~ Q(\eta ) = \exp (\sqrt{\tau }(\eta +k_0)).~~~~~~~~~~~~~~~~~~~~~~~~~~~~~~ \end{aligned}$$
  15 $$\begin{aligned} & {\textbf {Set 6:}}~\text {For}~~ \tau <0~~ \text {and} ~~\mu =0,~~\text {we have}~~ Q(\eta ) = \cos (\sqrt{-\tau }(\eta +k_0 ))+i \sin (\sqrt{-\tau }(\eta +k_0 )). \end{aligned}$$
  16 $$\begin{aligned} & {\textbf {Set 7:}}~\text {For}~~ \tau =0~~\text {and}~~ \mu >0,~~\text {we have}~ Q(\eta ) =\pm \frac{1}{\sqrt{\mu }(\eta +k_0 )}.~~~~~~~~~~~~~~~~~~~~~~~~~~~~~~~~~~ \end{aligned}$$
  17 $$\begin{aligned} & {\textbf {Set 8:}}~\text {For}~~ \tau =0 ~~\text {and}~~ \mu <0,~~\text {we have}~~ Q(\eta ) =\pm \frac{i}{\sqrt{-\mu }(\eta +k_0 )}.~~~~~~~~~~~~~~~~~~~~~~~~~~~~~~~ \end{aligned}$$
  **Form 2:** We consider that $Q(\eta )$ satisfies an ODE, taking the same process as previously utilized
  18 $$\begin{aligned} \frac{dQ}{d\eta } = \tau +\mu \nabla ^2 ~~~\tau ,\mu \in \Re . \end{aligned}$$

Applying the balance principle formula to Eq. (7) finds *n*. Inserting Eq. (8) into Eq. (7), we get a set of nonlinear equations for $F_k$ ($k = 0, 1, 2, 3, \ldots , n$), with the solutions set that permits Eq. (18).

19 $$\begin{aligned} & {\textbf {Set 1:}}~\text {For}~~ \tau \mu >0,~~\text {we have}~~Q(\eta ) =sgn(\tau ) \sqrt{\frac{\tau }{\mu }}\tan (\sqrt{\tau \eta } (\eta +k_0 ).~~~~~~~ \end{aligned}$$

20 $$\begin{aligned} & {\textbf {Set 2:}}~\text {For}~~ \tau \mu >0,~~\text {we have}~~ Q(\eta ) =-sgn(\tau ) \sqrt{\frac{\tau }{\mu }}\cot (\sqrt{\tau \mu } (\eta +k_0 ).~~~~~ \end{aligned}$$

21 $$\begin{aligned} & {\textbf {Set 3:}}~\text {For}~~ \tau \mu <0,~~\text {we have}~~ Q(\eta ) =sgn(\tau ) \sqrt{\frac{\tau }{-\mu }}\tanh (\sqrt{-\tau \mu } (\eta +k_0 ).~ \end{aligned}$$

22 $$\begin{aligned} & {\textbf {Set 4:}}~\text {For}~~ \tau \mu <0,~~\text {we have}~~ Q(\eta ) =sgn(\tau ) \sqrt{\frac{\tau }{-\mu }}\coth (\sqrt{-\tau \mu } (\eta +k_0 ).~ \end{aligned}$$

23 $$\begin{aligned} & {\textbf {Set 5:}}~\text {For}~~ \tau =0~~ \text {and} ~~\mu >0,~~\text {we have}~~ Q(\eta ) = - \frac{1}{\mu (\eta +k_0 )}.~~~~~~~~~~~~~~ \end{aligned}$$

24 $$\begin{aligned} & {\textbf {Set 6:}}~\text {For}~~ \tau \in \Re ~\text {and}~~ \mu =0,~~\text {we have}~~ Q(\eta ) = \tau (\eta +k_0 ).~~~~~~~~~~~~~~~~~ \end{aligned}$$

#### Application of the new EHFM

**Form-1.**

The solutions obtained via the new EHFM is presented below:

25 $$\begin{aligned} {\left\{ \begin{array}{ll}{\begin{matrix} p(\eta )=a_{0}+\sum _{h=1}^{n}a_{h}(Q(\eta ))^{h}.\\ q(\eta )=b_{0}+\sum _{h=1}^{n}b_{h}(Q(\eta ))^{h}.\\ r(\eta )=c_{0}+\sum _{h=1}^{n}c_{h}(Q(\eta ))^{h}. \end{matrix}} \end{array}\right. } \end{aligned}$$

For $n=1$, the above solutions of Eq. (25) turn into:

26 $$\begin{aligned} {\left\{ \begin{array}{ll} p(\eta )=a_0+a_1 Q(\eta ).\\ q(\eta )=b_0+b_1 Q(\eta ).\\ r(\eta )=b_0+b_1 Q(\eta ). \end{array}\right. } \end{aligned}$$

By putting Eq. (26) with Eq. (9) into Eq. (4) and setting coefficients of like powers of $Q(\eta )$ equal to 0, with the help of computational software Mathematica. We achieve solution sets.

**Set-1.**

$$\begin{aligned} a_0=0,~a_1=a_1,~b_1=\sqrt{a_1 c_1},~b_0=0,~c_0=0,~c_1=\sqrt{\mu }-a_1,~\sigma =-\sqrt{\tau -\omega }. \end{aligned}$$

Many new types of solutions to Eq. (2) are established and discussed in detail:

**(1)** For $$\tau>0,~\text {and}~~ \mu >0$$,

- Solitary wave profiles

27 $$\begin{aligned} \Upsilon _{+1}(x,t)=\Bigg \lbrace -\frac{a_1 \sqrt{\tau } \exp \left( i \left( \frac{\omega \left( \frac{1}{\Gamma (\varrho )}+t\right) ^{\varrho }}{\varrho }-\frac{\sqrt{\tau -\omega } \left( \frac{1}{\Gamma (\varrho )}+x\right) ^{\varrho }}{\varrho }\right) \right) \text {csch}\left( \frac{\sqrt{\tau } \left( \varrho k_0+2 \sqrt{\tau -\omega } \left( \frac{1}{\Gamma (\varrho )}+t\right) ^{\varrho }+\left( \frac{1}{\Gamma (\varrho )}+x\right) ^{\varrho }\right) }{\varrho }\right) }{\sqrt{\mu }}\Bigg \rbrace . \end{aligned}$$

28 $$\begin{aligned} \Upsilon _{0}(x,t)=\Bigg \lbrace -\frac{\sqrt{\tau } \sqrt{a_1 \left( \sqrt{\mu }-a_1\right) } \exp \left( i \left( \frac{\omega \left( \frac{1}{\Gamma (\varrho )}+t\right) ^{\varrho }}{\varrho }-\frac{\sqrt{\tau -\omega } \left( \frac{1}{\Gamma (\varrho )}+x\right) ^{\varrho }}{\varrho }\right) \right) \text {csch}\left( \frac{\sqrt{\tau } \left( \varrho k_0+2 \sqrt{\tau -\omega } \left( \frac{1}{\Gamma (\varrho )}+t\right) ^{\varrho }+\left( \frac{1}{\Gamma (\varrho )}+x\right) ^{\varrho }\right) }{\varrho }\right) }{\sqrt{\mu }}\Bigg \rbrace .\end{aligned}$$

29 $$\begin{aligned} \Upsilon _{-1}(x,t)=\Bigg \lbrace \frac{\sqrt{\tau } \left( a_1-\sqrt{\mu }\right) \exp \left( i \left( \frac{\omega \left( \frac{1}{\Gamma (\varrho )}+t\right) ^{\varrho }}{\varrho }-\frac{\sqrt{\tau -\omega } \left( \frac{1}{\Gamma (\varrho )}+x\right) ^{\varrho }}{\varrho }\right) \right) \text {csch}\left( \frac{\sqrt{\tau } \left( \varrho k_0+2 \sqrt{\tau -\omega } \left( \frac{1}{\Gamma (\varrho )}+t\right) ^{\varrho }+\left( \frac{1}{\Gamma (\varrho )}+x\right) ^{\varrho }\right) }{\varrho }\right) }{\sqrt{\mu }}\Bigg \rbrace . \end{aligned}$$

**(2)** For $$\tau <0,~\text {and}~~ \mu >0$$,

- Trigonometric structures

30 $$\begin{aligned} \Upsilon _{+1}(x,t)=\Bigg \lbrace \frac{a_1 \sqrt{-\tau } \exp \left( i \left( \frac{\omega \left( \frac{1}{\Gamma (\varrho )}+t\right) ^{\varrho }}{\varrho }-\frac{\sqrt{\tau -\omega } \left( \frac{1}{\Gamma (\varrho )}+x\right) ^{\varrho }}{\varrho }\right) \right) \text {sec}\left( \frac{\sqrt{-\tau } \left( \varrho k_0+2 \sqrt{\tau -\omega } \left( \frac{1}{\Gamma (\varrho )}+t\right) ^{\varrho }+\left( \frac{1}{\Gamma (\varrho )}+x\right) ^{\varrho }\right) }{\varrho }\right) }{\sqrt{\mu }}\Bigg \rbrace . \end{aligned}$$

31 $$\begin{aligned} \Upsilon _{0}(x,t)=\Bigg \lbrace \frac{\sqrt{-\tau } \sqrt{a_1 \left( \sqrt{\mu }-a_1\right) } \exp \left( i \left( \frac{\omega \left( \frac{1}{\Gamma (\varrho )}+t\right) ^{\varrho }}{\varrho }-\frac{\sqrt{\tau -\omega } \left( \frac{1}{\Gamma (\varrho )}+x\right) ^{\varrho }}{\varrho }\right) \right) \text {sec}\left( \frac{\sqrt{-\tau } \left( \varrho k_0+2 \sqrt{\tau -\omega } \left( \frac{1}{\Gamma (\varrho )}+t\right) ^{\varrho }+\left( \frac{1}{\Gamma (\varrho )}+x\right) ^{\varrho }\right) }{\varrho }\right) }{\sqrt{\mu }}\Bigg \rbrace .\end{aligned}$$

32 $$\begin{aligned} \Upsilon _{-1}(x,t)=\Bigg \lbrace \frac{\sqrt{-\tau } \left( \sqrt{\mu }-a_1\right) \exp \left( i \left( \frac{\omega \left( \frac{1}{\Gamma (\varrho )}+t\right) ^{\varrho }}{\varrho }-\frac{\sqrt{\tau -\omega } \left( \frac{1}{\Gamma (\varrho )}+x\right) ^{\varrho }}{\varrho }\right) \right) \text {sec}\left( \frac{\sqrt{-\tau } \left( \varrho k_0+2 \sqrt{\tau -\omega } \left( \frac{1}{\Gamma (\varrho )}+t\right) ^{\varrho }+\left( \frac{1}{\Gamma (\varrho )}+x\right) ^{\varrho }\right) }{\varrho }\right) }{\sqrt{\mu }}\Bigg \rbrace .~~~~~~~ \end{aligned}$$

**(3)** For $$\tau >0,~\text {and}~~ \mu <0$$,

- Bright wave solutions

33 $$\begin{aligned} \Upsilon _{+1}(x,t)=\Bigg \lbrace \frac{a_1 \sqrt{\tau } \exp \left( i \left( \frac{\omega \left( \frac{1}{\Gamma (\varrho )}+t\right) ^{\varrho }}{\varrho }-\frac{\sqrt{\tau -\omega } \left( \frac{1}{\Gamma (\varrho )}+x\right) ^{\varrho }}{\varrho }\right) \right) \text {sech}\left( \frac{\sqrt{\tau } \left( \varrho k_0+2 \sqrt{\tau -\omega } \left( \frac{1}{\Gamma (\varrho )}+t\right) ^{\varrho }+\left( \frac{1}{\Gamma (\varrho )}+x\right) ^{\varrho }\right) }{\varrho }\right) }{\sqrt{-\mu }}\Bigg \rbrace .\end{aligned}$$

34 $$\begin{aligned} \Upsilon _{0}(x,t)=\Bigg \lbrace \frac{\sqrt{\tau } \sqrt{a_1 \left( \sqrt{\mu }-a_1\right) } \exp \left( i \left( \frac{\omega \left( \frac{1}{\Gamma (\varrho )}+t\right) ^{\varrho }}{\varrho }-\frac{\sqrt{\tau -\omega } \left( \frac{1}{\Gamma (\varrho )}+x\right) ^{\varrho }}{\varrho }\right) \right) \text {sech}\left( \frac{\sqrt{\tau } \left( \varrho k_0+2 \sqrt{\tau -\omega } \left( \frac{1}{\Gamma (\varrho )}+t\right) ^{\varrho }+\left( \frac{1}{\Gamma (\varrho )}+x\right) ^{\varrho }\right) }{\varrho }\right) }{\sqrt{-\mu }}\Bigg \rbrace .\end{aligned}$$

35 $$\begin{aligned} \Upsilon _{-1}(x,t)=\Bigg \lbrace \frac{\sqrt{\tau } \left( \sqrt{\mu }-a_1\right) \exp \left( i \left( \frac{\omega \left( \frac{1}{\Gamma (\varrho )}+t\right) ^{\varrho }}{\varrho }-\frac{\sqrt{\tau -\omega } \left( \frac{1}{\Gamma (\varrho )}+x\right) ^{\varrho }}{\varrho }\right) \right) \text {sech}\left( \frac{\sqrt{\tau } \left( \varrho k_0+2 \sqrt{\tau -\omega } \left( \frac{1}{\Gamma (\varrho )}+t\right) ^{\varrho }+\left( \frac{1}{\Gamma (\varrho )}+x\right) ^{\varrho }\right) }{\varrho }\right) }{\sqrt{-\mu }}\Bigg \rbrace .~~~~~~~ \end{aligned}$$

**(4)** For $$\tau <0,~\text {and}~~ \mu >0$$,

- Trigonometric solutions

36 $$\begin{aligned} \Upsilon _{+1}(x,t)=\Bigg \lbrace \frac{a_1 \sqrt{-\tau } \exp \left( i \left( \frac{\omega \left( \frac{1}{\Gamma (\varrho )}+t\right) ^{\varrho }}{\varrho }-\frac{\sqrt{\tau -\omega } \left( \frac{1}{\Gamma (\varrho )}+x\right) ^{\varrho }}{\varrho }\right) \right) \text {csc}\left( \frac{\sqrt{-\tau } \left( \varrho k_0+2 \sqrt{\tau -\omega } \left( \frac{1}{\Gamma (\varrho )}+t\right) ^{\varrho }+\left( \frac{1}{\Gamma (\varrho )}+x\right) ^{\varrho }\right) }{\varrho }\right) }{\sqrt{\mu }}\Bigg \rbrace .\end{aligned}$$

37 $$\begin{aligned} \Upsilon _{0}(x,t)=\Bigg \lbrace \frac{\sqrt{-\tau } \sqrt{a_1 \left( \sqrt{\mu }-a_1\right) } \exp \left( i \left( \frac{\omega \left( \frac{1}{\Gamma (\varrho )}+t\right) ^{\varrho }}{\varrho }-\frac{\sqrt{\tau -\omega } \left( \frac{1}{\Gamma (\varrho )}+x\right) ^{\varrho }}{\varrho }\right) \right) \text {csc}\left( \frac{\sqrt{-\tau } \left( \varrho k_0+2 \sqrt{\tau -\omega } \left( \frac{1}{\Gamma (\varrho )}+t\right) ^{\varrho }+\left( \frac{1}{\Gamma (\varrho )}+x\right) ^{\varrho }\right) }{\varrho }\right) }{\sqrt{\mu }}\Bigg \rbrace .\end{aligned}$$

38 $$\begin{aligned} \Upsilon _{-1}(x,t)=\Bigg \lbrace \frac{\sqrt{-\tau } \left( \sqrt{\mu }-a_1\right) \exp \left( i \left( \frac{\omega \left( \frac{1}{\Gamma (\varrho )}+t\right) ^{\varrho }}{\varrho }-\frac{\sqrt{\tau -\omega } \left( \frac{1}{\Gamma (\varrho )}+x\right) ^{\varrho }}{\varrho }\right) \right) \text {csc}\left( \frac{\sqrt{-\tau } \left( \varrho k_0+2 \sqrt{\tau -\omega } \left( \frac{1}{\Gamma (\varrho )}+t\right) ^{\varrho }+\left( \frac{1}{\Gamma (\varrho )}+x\right) ^{\varrho }\right) }{\varrho }\right) }{\sqrt{\mu }}\Bigg \rbrace .~~~~~~ \end{aligned}$$

**(5)** For $$\tau >0,~\text {and}~~ \mu =0$$,

- Exponential form solutions

39 $$\begin{aligned} & \Upsilon _{+1}(x,t)=\Bigg \lbrace a_1 \exp \left( \frac{\sqrt{\tau } \left( \varrho k_0+2 \sqrt{\tau -\omega } \left( \frac{1}{\Gamma (\varrho )}+t\right) ^{\varrho }+\left( \frac{1}{\Gamma (\varrho )}+x\right) ^{\varrho }\right) }{\varrho }+i \left( \frac{\omega \left( \frac{1}{\Gamma (\varrho )}+t\right) ^{\varrho }}{\varrho }-\frac{\sqrt{\tau -\omega } \left( \frac{1}{\Gamma (\varrho )}+x\right) ^{\varrho }}{\varrho }\right) \right) \Bigg \rbrace .\end{aligned}$$

40 $$\begin{aligned} & \Upsilon _{0}(x,t)=\Bigg \lbrace \sqrt{-a_1^2} \exp \left( \frac{\sqrt{\tau } \left( \varrho k_0+2 \sqrt{\tau -\omega } \left( \frac{1}{\Gamma (\varrho )}+t\right) ^{\varrho }+\left( \frac{1}{\Gamma (\varrho )}+x\right) ^{\varrho }\right) }{\varrho }+i \left( \frac{\omega \left( \frac{1}{\Gamma (\varrho )}+t\right) ^{\varrho }}{\varrho }-\frac{\sqrt{\tau -\omega } \left( \frac{1}{\Gamma (\varrho )}+x\right) ^{\varrho }}{\varrho }\right) \right) \Bigg \rbrace .\end{aligned}$$

41 $$\begin{aligned} & \Upsilon _{-1}(x,t)=\Bigg \lbrace a_1 \left( -\exp \left( \frac{\sqrt{\tau } \left( \varrho k_0+2 \sqrt{\tau -\omega } \left( \frac{1}{\Gamma (\varrho )}+t\right) ^{\varrho }+\left( \frac{1}{\Gamma (\varrho )}+x\right) ^{\varrho }\right) }{\varrho }+i \left( \frac{\omega \left( \frac{1}{\Gamma (\varrho )}+t\right) ^{\varrho }}{\varrho }-\frac{\sqrt{\tau -\omega } \left( \frac{1}{\Gamma (\varrho )}+x\right) ^{\varrho }}{\varrho }\right) \right) \right) \Bigg \rbrace . \end{aligned}$$

**(6)** For $$\tau <0,~\text {and}~~ \mu =0$$,

- Exponential form solutions

42 $$\begin{gathered} \Upsilon _{{ + 1}} (x,t) = \{ a_{1} \left( \begin{gathered} \cos \left( {\frac{{\sqrt { - \tau } \left( {{\varrho }k_{0} + 2\sqrt {\tau - \omega } \left( {\frac{1}{{\Gamma ({\varrho })}} + t} \right)^{{\varrho }} + \left( {\frac{1}{{\Gamma ({\varrho })}} + x} \right)^{{\varrho }} } \right)}}{{\varrho }}} \right) \hfill \\ + i\sin \left( {\frac{{\sqrt { - \tau } \left( {{\varrho }k_{0} + 2\sqrt {\tau - \omega } \left( {\frac{1}{{\Gamma ({\varrho })}} + t} \right)^{{\varrho }} + \left( {\frac{1}{{\Gamma ({\varrho })}} + x} \right)^{{\varrho }} } \right)}}{{\varrho }}} \right) \hfill \\ \end{gathered} \right)\} \hfill \\ \times \exp \left( {i\left( {\frac{{\omega \left( {\frac{1}{{\Gamma ({\varrho })}} + t} \right)^{{\varrho }} }}{{\varrho }} - \frac{{\sqrt {\tau - \omega } \left( {\frac{1}{{\Gamma ({\varrho })}} + x} \right)^{{\varrho }} }}{{\varrho }}} \right)} \right). \hfill \\ \end{gathered}$$

43 $$\begin{gathered} \Upsilon _{0} (x,t) = \{ \sqrt { - a_{1}^{2} } \left( \begin{gathered} \cos \left( {\frac{{\sqrt { - \tau } \left( {{\varrho }k_{0} + 2\sqrt {\tau - \omega } \left( {\frac{1}{{\Gamma ({\varrho })}} + t} \right)^{{\varrho }} + \left( {\frac{1}{{\Gamma ({\varrho })}} + x} \right)^{{\varrho }} } \right)}}{{\varrho }}} \right) \hfill \\ + i\sin \left( {\frac{{\sqrt { - \tau } \left( {{\varrho }k_{0} + 2\sqrt {\tau - \omega } \left( {\frac{1}{{\Gamma ({\varrho })}} + t} \right)^{{\varrho }} + \left( {\frac{1}{{\Gamma ({\varrho })}} + x} \right)^{{\varrho }} } \right)}}{{\varrho }}} \right) \hfill \\ \end{gathered} \right)\} \hfill \\ \times \exp \left( {i\left( {\frac{{\omega \left( {\frac{1}{{\Gamma ({\varrho })}} + t} \right)^{{\varrho }} }}{{\varrho }} - \frac{{\sqrt {\tau - \omega } \left( {\frac{1}{{\Gamma ({\varrho })}} + x} \right)^{{\varrho }} }}{{\varrho }}} \right)} \right). \hfill \\ \end{gathered}$$

44 $$\begin{gathered} \Upsilon _{{ - 1}} (x,t) = \{ - a_{1} \left( \begin{gathered} \cos \left( {\frac{{\sqrt { - \tau } \left( {{\varrho }k_{0} + 2\sqrt {\tau - \omega } \left( {\frac{1}{{\Gamma ({\varrho })}} + t} \right)^{{\varrho }} + \left( {\frac{1}{{\Gamma ({\varrho })}} + x} \right)^{{\varrho }} } \right)}}{{\varrho }}} \right) \hfill \\ + i\sin \left( {\frac{{\sqrt { - \tau } \left( {{\varrho }k_{0} + 2\sqrt {\tau - \omega } \left( {\frac{1}{{\Gamma ({\varrho })}} + t} \right)^{{\varrho }} + \left( {\frac{1}{{\Gamma ({\varrho })}} + x} \right)^{{\varrho }} } \right)}}{{\varrho }}} \right) \hfill \\ \end{gathered} \right)\} \hfill \\ \times \left( {\exp \left( {i\left( {\frac{{\omega \left( {\frac{1}{{\Gamma ({\varrho })}} + t} \right)^{{\varrho }} }}{{\varrho }} - \frac{{\sqrt {\tau - \omega } \left( {\frac{1}{{\Gamma ({\varrho })}} + x} \right)^{{\varrho }} }}{{\varrho }}} \right)} \right)} \right). \hfill \\ \end{gathered}$$

**(7)** For $$\tau =0,~\text {and}~~ \mu >0$$,

- Rational function solutions

45 $$\begin{aligned} \Upsilon _{+1}(x,t)=\Bigg \lbrace \frac{ i a_1 \varrho \exp \left( i \left( \frac{\omega \left( \frac{1}{\Gamma (\varrho )}+t\right) ^{\varrho }}{\varrho }-\frac{\sqrt{-\omega } \left( \frac{1}{\Gamma (\varrho )}+x\right) ^{\varrho }}{\varrho }\right) \right) }{\sqrt{-\mu } \left( \varrho k_0+2 \sqrt{-\omega } \left( \frac{1}{\Gamma (\varrho )}+t\right) ^{\varrho }+\left( \frac{1}{\Gamma (\varrho )}+x\right) ^{\varrho }\right) }\Bigg \rbrace .\end{aligned}$$

46 $$\begin{aligned} \Upsilon _{0}(x,t)=\Bigg \lbrace \frac{i \varrho \sqrt{a_1 \left( \sqrt{\mu }-a_1\right) } \exp \left( i \left( \frac{\omega \left( \frac{1}{\Gamma (\varrho )}+t\right) ^{\varrho }}{\varrho }-\frac{\sqrt{-\omega } \left( \frac{1}{\Gamma (\varrho )}+x\right) ^{\varrho }}{\varrho }\right) \right) }{\sqrt{-\mu } \left( \varrho k_0+2 \sqrt{-\omega } \left( \frac{1}{\Gamma (\varrho )}+t\right) ^{\varrho }+\left( \frac{1}{\Gamma (\varrho )}+x\right) ^{\varrho }\right) }\Bigg \rbrace .\end{aligned}$$

47 $$\begin{aligned} \Upsilon _{-1}(x,t)=\Bigg \lbrace \frac{i \varrho \left( \sqrt{\mu }-a_1\right) \exp \left( i \left( \frac{\omega \left( \frac{1}{\Gamma (\varrho )}+t\right) ^{\varrho }}{\varrho }-\frac{\sqrt{-\omega } \left( \frac{1}{\Gamma (\varrho )}+x\right) ^{\varrho }}{\varrho }\right) \right) }{\sqrt{-\mu } \left( \varrho k_0+2 \sqrt{-\omega } \left( \frac{1}{\Gamma (\varrho )}+t\right) ^{\varrho }+\left( \frac{1}{\Gamma (\varrho )}+x\right) ^{\varrho }\right) }\Bigg \rbrace .~~~~~~~~~~~~~ \end{aligned}$$

**Form-2.**

Inserting Eq. (26) along with Eq. (18) into Eq. (4) and substituting $Q(\eta )$ to zero yeilds, a system of algebraic equations. Solving this nonlinear system exposes some distinct families of solutions.

**Set-1.**

$$\begin{gathered} a_{0} = - \frac{{i\sqrt \tau \left( {\sqrt {\mu ^{2} - 4b_{1}^{2} } + \mu } \right)}}{{2\sqrt \mu }},~b_{0} = - \frac{{ib_{1} \sqrt \tau }}{{\sqrt \mu }} \hfill \\ ,~a_{1} = \frac{1}{2}\left( {\sqrt {\mu ^{2} - 4b_{1}^{2} } - \mu } \right),~c_{0} = \frac{{i\sqrt \tau \left( {\sqrt {\mu ^{2} - 4b_{1}^{2} } - \mu } \right)}}{{2\sqrt \mu }},~ \hfill \\ c_{1} = \frac{1}{2}\left( { - \sqrt {\mu ^{2} - 4b_{1}^{2} } - \mu } \right),~\sigma = \sqrt {2\mu \tau - \omega } . \hfill \\ \hfill \\ \end{gathered}.$$

**(1)** For $\tau \mu >0$, a variety of exact solutions are obtained.

- The trigonometric solutions

48 $$\begin{aligned} & \Upsilon _{+1}(x,t)=\Bigg \lbrace \Bbbk \frac{\sqrt{\tau } \left( \left( \sqrt{\mu ^2-4 b_1^2}-\mu \right) \tan \left( \frac{\sqrt{\mu \tau } \left( \varrho k_0-2 \sqrt{2 \mu \tau -\omega } \left( \frac{1}{\Gamma (\varrho )}+t\right) ^{\varrho }+\left( \frac{1}{\Gamma (\varrho )}+x\right) ^{\varrho }\right) }{\varrho }\right) -i \left( \sqrt{\mu ^2-4 b_1^2}+\mu \right) \right) }{2 \sqrt{\mu }}\Bigg \rbrace \nonumber \\ & \times \exp \left( i \left( \frac{\omega \left( \frac{1}{\Gamma (\varrho )}+t\right) ^{\varrho }}{\varrho }+\frac{\sqrt{2 \mu \tau -\omega } \left( \frac{1}{\Gamma (\varrho )}+x\right) ^{\varrho }}{\varrho }\right) \right) .\end{aligned}$$

49 $$\begin{aligned} & \Upsilon _{0}(x,t)=\Bigg \lbrace \Bbbk \frac{b_1 \sqrt{\tau } \left( \tan \left( \frac{\sqrt{\mu \tau } \left( \varrho k_0-2 \sqrt{2 \mu \tau -\omega } \left( \frac{1}{\Gamma (\varrho )}+t\right) ^{\varrho }+\left( \frac{1}{\Gamma (\varrho )}+x\right) ^{\varrho }\right) }{\varrho }\right) -i\right) }{\sqrt{\mu }}\Bigg \rbrace \nonumber \\ & \times \exp \left( i \left( \frac{\omega \left( \frac{1}{\Gamma (\varrho )}+t\right) ^{\varrho }}{\varrho }+\frac{\sqrt{2 \mu \tau -\omega } \left( \frac{1}{\Gamma (\varrho )}+x\right) ^{\varrho }}{\varrho }\right) \right) . \end{aligned}$$

50 $$\begin{aligned} & \Upsilon _{-1}(x,t)=\Bigg \lbrace \Bbbk \frac{\sqrt{\tau } \left( -\left( \sqrt{\mu ^2-4 b_1^2}+\mu \right) \tan \left( \frac{\sqrt{\mu \tau } \left( \varrho k_0-2 \sqrt{2 \mu \tau -\omega } \left( \frac{1}{\Gamma (\varrho )}+t\right) ^{\varrho }+\left( \frac{1}{\Gamma (\varrho )}+x\right) ^{\varrho }\right) }{\varrho }\right) +i \left( \sqrt{\mu ^2-4 b_1^2}-\mu \right) \right) }{2 \sqrt{\mu }}\Bigg \rbrace \nonumber \\ & \times \exp \left( i \left( \frac{\omega \left( \frac{1}{\Gamma (\varrho )}+t\right) ^{\varrho }}{\varrho }+\frac{\sqrt{2 \mu \tau -\omega } \left( \frac{1}{\Gamma (\varrho )}+x\right) ^{\varrho }}{\varrho }\right) \right) . \end{aligned}$$

51 $$\begin{aligned} & \Upsilon _{+1}(x,t)=\Bigg \lbrace -\Bbbk \frac{\sqrt{\tau } \left( \left( \sqrt{\mu ^2-4 b_1^2}-\mu \right) \cot \left( \frac{\sqrt{\mu \tau } \left( \varrho k_0-2 \sqrt{2 \mu \tau -\omega } \left( \frac{1}{\Gamma (\varrho )}+t\right) ^{\varrho }+\left( \frac{1}{\Gamma (\varrho )}+x\right) ^{\varrho }\right) }{\varrho }\right) +i \left( \sqrt{\mu ^2-4 b_1^2}+\mu \right) \right) }{2 \sqrt{\mu }}\Bigg \rbrace \nonumber \\ & \times \exp \left( i \left( \frac{\omega \left( \frac{1}{\Gamma (\varrho )}+t\right) ^{\varrho }}{\varrho }+\frac{\sqrt{2 \mu \tau -\omega } \left( \frac{1}{\Gamma (\varrho )}+x\right) ^{\varrho }}{\varrho }\right) \right) .\end{aligned}$$

52 $$\begin{aligned} & \Upsilon _{0}(x,t)=\Bigg \lbrace -\Bbbk \frac{b_1 \sqrt{\tau } \left( \cot \left( \frac{\sqrt{\mu \tau } \left( \varrho k_0-2 \sqrt{2 \mu \tau -\omega } \left( \frac{1}{\Gamma (\varrho )}+t\right) ^{\varrho }+\left( \frac{1}{\Gamma (\varrho )}+x\right) ^{\varrho }\right) }{\varrho }\right) +i\right) }{\sqrt{\mu }}\Bigg \rbrace \nonumber \\ & \times \exp \left( i \left( \frac{\omega \left( \frac{1}{\Gamma (\varrho )}+t\right) ^{\varrho }}{\varrho }+\frac{\sqrt{2 \mu \tau -\omega } \left( \frac{1}{\Gamma (\varrho )}+x\right) ^{\varrho }}{\varrho }\right) \right) .\end{aligned}$$

53 $$\begin{aligned} & \Upsilon _{-1}(x,t)=\Bigg \lbrace \Bbbk \frac{\sqrt{\tau } \left( \left( \sqrt{\mu ^2-4 b_1^2}+\mu \right) \cot \left( \frac{\sqrt{\mu \tau } \left( \varrho k_0-2 \sqrt{2 \mu \tau -\omega } \left( \frac{1}{\Gamma (\varrho )}+t\right) ^{\varrho }+\left( \frac{1}{\Gamma (\varrho )}+x\right) ^{\varrho }\right) }{\varrho }\right) +i \left( \sqrt{\mu ^2-4 b_1^2}-\mu \right) \right) }{2 \sqrt{\mu }}\Bigg \rbrace \nonumber \\ & \times \exp \left( i \left( \frac{\omega \left( \frac{1}{\Gamma (\varrho )}+t\right) ^{\varrho }}{\varrho }+\frac{\sqrt{2 \mu \tau -\omega } \left( \frac{1}{\Gamma (\varrho )}+x\right) ^{\varrho }}{\varrho }\right) \right) . \end{aligned}$$

**(2)** For $\tau \mu <0$, various exact solutions are constructed.

- The dark wave solutions

54 $$\begin{aligned} & \Upsilon _{+1}(x,t)=\Bigg \lbrace \Bbbk \frac{1}{2} \sqrt{\tau } \left( \frac{\left( \sqrt{\mu ^2-4 b_1^2}-\mu \right) \tanh \left( \frac{\sqrt{-\mu \tau } \left( \varrho k_0-2 \sqrt{2 \mu \tau -\omega } \left( \frac{1}{\Gamma (\varrho )}+t\right) ^{\varrho }+\left( \frac{1}{\Gamma (\varrho )}+x\right) ^{\varrho }\right) }{\varrho }\right) }{\sqrt{-\mu }}-\frac{i \left( \sqrt{\mu ^2-4 b_1^2}+\mu \right) }{\sqrt{\mu }}\right) \Bigg \rbrace \nonumber \\ & \times \exp \left( i \left( \frac{\omega \left( \frac{1}{\Gamma (\varrho )}+t\right) ^{\varrho }}{\varrho }+\frac{\sqrt{2 \mu \tau -\omega } \left( \frac{1}{\Gamma (\varrho )}+x\right) ^{\varrho }}{\varrho }\right) \right) .\end{aligned}$$

55 $$\begin{aligned} & \Upsilon _{0}(x,t)=\Bigg \lbrace \Bbbk \frac{b_1 \sqrt{\tau } \left( \sqrt{\mu } \tanh \left( \frac{\sqrt{-\mu \tau } \left( \varrho k_0-2 \sqrt{2 \mu \tau -\omega } \left( \frac{1}{\Gamma (\varrho )}+t\right) ^{\varrho }+\left( \frac{1}{\Gamma (\varrho )}+x\right) ^{\varrho }\right) }{\varrho }\right) -i \sqrt{-\mu }\right) }{\sqrt{-\mu ^2}}\Bigg \rbrace \nonumber \\ & \times \exp \left( i \left( \frac{\omega \left( \frac{1}{\Gamma (\varrho )}+t\right) ^{\varrho }}{\varrho }+\frac{\sqrt{2 \mu \tau -\omega } \left( \frac{1}{\Gamma (\varrho )}+x\right) ^{\varrho }}{\varrho }\right) \right) .\end{aligned}$$

56 $$\begin{aligned} & \Upsilon _{-1}(x,t)=\Bigg \lbrace - \Bbbk \frac{\sqrt{\tau } \left( \sqrt{\mu } \left( \sqrt{\mu ^2-4 b_1^2}+\mu \right) \tanh \left( \frac{\sqrt{-\mu \tau } \left( \varrho k_0-2 \sqrt{2 \mu \tau -\omega } \left( \frac{1}{\Gamma (\varrho )}+t\right) ^{\varrho }+\left( \frac{1}{\Gamma (\varrho )}+x\right) ^{\varrho }\right) }{\varrho }\right) +i \sqrt{-\mu } \left( \mu -\sqrt{\mu ^2-4 b_1^2}\right) \right) }{2 \sqrt{-\mu ^2}}\Bigg \rbrace \nonumber \\ & \times \exp \left( i \left( \frac{\omega \left( \frac{1}{\Gamma (\varrho )}+t\right) ^{\varrho }}{\varrho }+\frac{\sqrt{2 \mu \tau -\omega } \left( \frac{1}{\Gamma (\varrho )}+x\right) ^{\varrho }}{\varrho }\right) \right) . \end{aligned}$$

- The singular optical solutions

57 $$\begin{aligned} & \Upsilon _{+1}(x,t)=\Bigg \lbrace \Bbbk \frac{1}{2} \sqrt{\tau } \left( \frac{\left( \sqrt{\mu ^2-4 b_1^2}-\mu \right) \coth \left( \frac{\sqrt{-\mu \tau } \left( \varrho k_0-2 \sqrt{2 \mu \tau -\omega } \left( \frac{1}{\Gamma (\varrho )}+t\right) ^{\varrho }+\left( \frac{1}{\Gamma (\varrho )}+x\right) ^{\varrho }\right) }{\varrho }\right) }{\sqrt{-\mu }}-\frac{i \left( \sqrt{\mu ^2-4 b_1^2}+\mu \right) }{\sqrt{\mu }}\right) \Bigg \rbrace \nonumber \\ & \times \exp \left( i \left( \frac{\omega \left( \frac{1}{\Gamma (\varrho )}+t\right) ^{\varrho }}{\varrho }+\frac{\sqrt{2 \mu \tau -\omega } \left( \frac{1}{\Gamma (\varrho )}+x\right) ^{\varrho }}{\varrho }\right) \right) .\end{aligned}$$

58 $$\begin{aligned} & \Upsilon _{0}(x,t)=\Bigg \lbrace \Bbbk \frac{b_1 \sqrt{\tau } \left( \sqrt{\mu } \coth \left( \frac{\sqrt{-\mu \tau } \left( \varrho k_0-2 \sqrt{2 \mu \tau -\omega } \left( \frac{1}{\Gamma (\varrho )}+t\right) ^{\varrho }+\left( \frac{1}{\Gamma (\varrho )}+x\right) ^{\varrho }\right) }{\varrho }\right) -i \sqrt{-\mu }\right) }{\sqrt{-\mu ^2}}\Bigg \rbrace \nonumber \\ & \times \exp \left( i \left( \frac{\omega \left( \frac{1}{\Gamma (\varrho )}+t\right) ^{\varrho }}{\varrho }+\frac{\sqrt{2 \mu \tau -\omega } \left( \frac{1}{\Gamma (\varrho )}+x\right) ^{\varrho }}{\varrho }\right) \right) .\end{aligned}$$

59 $$\begin{aligned} & \Upsilon _{-1}(x,t)=\Bigg \lbrace -\Bbbk \frac{\sqrt{\tau } \left( \sqrt{\mu } \left( \sqrt{\mu ^2-4 b_1^2}+\mu \right) \coth \left( \frac{\sqrt{-\mu \tau } \left( \varrho k_0-2 \sqrt{2 \mu \tau -\omega } \left( \frac{1}{\Gamma (\varrho )}+t\right) ^{\varrho }+\left( \frac{1}{\Gamma (\varrho )}+x\right) ^{\varrho }\right) }{\varrho }\right) +i \sqrt{-\mu } \left( \mu -\sqrt{\mu ^2-4 b_1^2}\right) \right) }{2 \sqrt{-\mu ^2}}\Bigg \rbrace \nonumber \\ & \times \exp \left( i \left( \frac{\omega \left( \frac{1}{\Gamma (\varrho )}+t\right) ^{\varrho }}{\varrho }+\frac{\sqrt{2 \mu \tau -\omega } \left( \frac{1}{\Gamma (\varrho )}+x\right) ^{\varrho }}{\varrho }\right) \right) . \end{aligned}$$

For the above solutions $\Bbbk =sgn(\tau )$.

### Solutions via unified method

The unified method yields the following solution:

60 $$\begin{aligned} {\left\{ \begin{array}{ll} p(\eta )=a_{0}+\sum _{u=1}^{n}(a_{u} Q(\eta )^u+ f_{u}Q(\eta )^{-u}).\\ q(\eta )=b_{0}+\sum _{u=1}^{n}(b_{u} Q(\eta )^u+ g_{u}Q(\eta )^{-u}).\\ r(\eta )=c_{0}+\sum _{u=1}^{n}(c_{u} Q(\eta )^u+ h_{u}Q(\eta )^{-u}). \end{array}\right. } \end{aligned}$$

Furthermore, $Q(\eta )$ fulfils the following Riccati ODE:

61 $$\begin{aligned} Q'(\eta )= Q^2(\eta )+\varpi . \end{aligned}$$

Eq. (61) includes nine different kinds of solutions in three settings:

$${\textbf {Case-1: Hyperbolic function (when }}\varpi {\textbf { is negative)}}$$:

62 $$\begin{aligned} Q(\eta )= {\left\{ \begin{array}{ll} \frac{\sqrt{\varpi \left( -\left( A^2+B^2\right) \right) }-A \sqrt{-\varpi } \cosh \left( 2 \sqrt{-\varpi } (g+\eta )\right) }{A \sinh \left( 2 \sqrt{-\varpi } (g+\eta )\right) +B}.\\ -\frac{\sqrt{\varpi \left( -\left( A^2+B^2\right) \right) }-A \sqrt{-\varpi } \cosh \left( 2 \sqrt{-\varpi } (g+\eta )\right) }{A \sinh \left( 2 \sqrt{-\varpi } (g+\eta )\right) +B}.\\ \sqrt{-\varpi }-\frac{2 A \sqrt{-\varpi }}{A-\sinh \left( 2 \sqrt{-\varpi } (g+\eta )\right) +\cosh \left( 2 \sqrt{-\varpi } (g+\eta )\right) }.\\ \frac{2 A \sqrt{-\varpi }}{A-\sinh \left( 2 \sqrt{-\varpi } (g+\eta )\right) +\cosh \left( 2 \sqrt{-\varpi } (g+\eta )\right) }-\sqrt{-\varpi }. \end{array}\right. } \end{aligned}$$

$${\textbf {Case-2: Trigonometric function (when }}\varpi {\textbf { is positive)}}$$:

63 $$\begin{aligned} Q(\eta )= {\left\{ \begin{array}{ll} \frac{\sqrt{\varpi \left( A^2-B^2\right) }-A \sqrt{\varpi } \cos \left( 2 \sqrt{\varpi } (g+\eta )\right) }{A \sin \left( 2 \sqrt{\varpi } (g+\eta )\right) +B}.\\ -\frac{\sqrt{\varpi \left( A^2-B^2\right) }-A \sqrt{\varpi } \cos \left( 2 \sqrt{\varpi } (g+\eta )\right) }{A \sin \left( 2 \sqrt{\varpi } (g+\eta )\right) +B}.\\ i \sqrt{\varpi }-\frac{2 i A \sqrt{\varpi }}{A-i \sin \left( 2 \sqrt{\varpi } (g+\eta )\right) +\cos \left( 2 \sqrt{\varpi } (g+\eta )\right) }.\\ \frac{2 i A \sqrt{\varpi }}{A+i \sin \left( 2 \sqrt{\varpi } (g+\eta )\right) +\cos \left( 2 \sqrt{\varpi } (g+\eta )\right) }-i \sqrt{\varpi }. \end{array}\right. } \end{aligned}$$

$${\textbf {Case-3: Rational function (when }}\varpi {\textbf { =0)}}$$:

64 $$\begin{aligned} Q(\eta )=-\frac{1}{g+\eta }. \end{aligned}$$

When $A\ne 0$, *g* and *B* are arbitrary constants.

#### Implementation of the unified method

When $n=1$, Eq. (60)’s solution converts:

65 $$\begin{aligned} {\left\{ \begin{array}{ll}{\begin{matrix} p(\eta )=a_{0}+a_{1} Q(\eta )+ f_{1}Q(\eta )^{-1}.\\ q(\eta )=b_{0}+b_{1} Q(\eta )+ g_{1}Q(\eta )^{-1}.\\ r(\eta )=c_{0}+c_{1} Q(\eta )+ h_{1}Q(\eta )^{-1}. \end{matrix}} \end{array}\right. } \end{aligned}$$

Imposing Eq. (65) along with Eq. (61) into Eq. (4), we obtain sets of algebraic expressions that are nonlinear. Mathematica, a computer program, provides us with the following sets.

$$\begin{aligned} & \mathbf{{Set-1}}:\sigma =-\sqrt{2 \varpi -\omega },~a_0= 0,~a_1= 0,~b_0= 0,~b_1=0,~c_0= 0,~c_1= 0,~f_1= -\sqrt{\varpi ^2-g_1^2},~h_1= \sqrt{\varpi ^2-g_1^2}. \\ & \mathbf{{Set-2}}:\sigma =\sqrt{-\omega -4 \varpi },~a_0=0,~a_1=\frac{\sqrt{\varpi ^2-g_1^2}}{\varpi },~b_0=0,~b_1=\frac{g_1}{\varpi },~c_0=0,~c_1=-\frac{\sqrt{\varpi ^2-g_1^2}}{\varpi },\\ & f_1=\sqrt{\varpi ^2-g_1^2},~h_1=-\sqrt{\varpi ^2-g_1^2}. \\ & \mathbf{{Set-3}}:\sigma = \sqrt{-\omega -4 \varpi },~a_0= -\frac{\sqrt{\varpi ^2-4 g_1^2}+\varpi }{\sqrt{2} \sqrt{\varpi }},~a_1= \frac{\varpi -\sqrt{\varpi ^2-4 g_1^2}}{2 \varpi },~b_0= \frac{\sqrt{2} g_1}{\sqrt{\varpi }},~b_1= \frac{g_1}{\varpi },~\\ & c_0= \frac{\sqrt{\varpi ^2-4 g_1^2}-\varpi }{\sqrt{2} \sqrt{\varpi }},~c_1= \frac{\sqrt{\varpi ^2-4 g_1^2}+\varpi }{2 \varpi },~f_1=\frac{1}{2} \left( \varpi -\sqrt{\varpi ^2-4 g_1^2}\right) ,~h_1=\frac{1}{2} \left( \sqrt{\varpi ^2-4 g_1^2}+\varpi \right) . \end{aligned}$$

For** Set-1**,

** Case-1** **Hyperbolic solutions**:

If $$\varpi <0$$,

66 $$\begin{aligned} & \Upsilon _{+1}(x, t)_{1,1}=\frac{\sqrt{\varpi ^2-g_1^2} \exp \left( i \left( \frac{\omega \left( \frac{1}{\Gamma (\varrho )}+t\right) ^{\varrho }}{\varrho }-\frac{\sqrt{2 \varpi -\omega } \left( \frac{1}{\Gamma (\varrho )}+x\right) ^{\varrho }}{\varrho }\right) \right) \left( A \sinh \left( \frac{2 \sqrt{-\varpi } \left( \varrho g+2 \sqrt{2 \varpi -\omega } \left( \frac{1}{\Gamma (\varrho )}+t\right) ^{\varrho }+\left( \frac{1}{\Gamma (\varrho )}+x\right) ^{\varrho }\right) }{\varrho }\right) +B\right) }{\sqrt{\varpi \left( -\left( A^2+B^2\right) \right) }-A \sqrt{-\varpi } \cosh \left( \frac{2 \sqrt{-\varpi } \left( \varrho g+2 \sqrt{2 \varpi -\omega } \left( \frac{1}{\Gamma (\varrho )}+t\right) ^{\varrho }+\left( \frac{1}{\Gamma (\varrho )}+x\right) ^{\varrho }\right) }{\varrho }\right) }. \end{aligned}$$

67 $$\begin{aligned} & \Upsilon _{0}(x, t)_{1,1}=\frac{g_1 \exp \left( i \left( \frac{\omega \left( \frac{1}{\Gamma (\varrho )}+t\right) ^{\varrho }}{\varrho }-\frac{\sqrt{2 \varpi -\omega } \left( \frac{1}{\Gamma (\varrho )}+x\right) ^{\varrho }}{\varrho }\right) \right) \left( A \sinh \left( \frac{2 \sqrt{-\varpi } \left( \varrho g+2 \sqrt{2 \varpi -\omega } \left( \frac{1}{\Gamma (\varrho )}+t\right) ^{\varrho }+\left( \frac{1}{\Gamma (\varrho )}+x\right) ^{\varrho }\right) }{\varrho }\right) +B\right) }{\sqrt{\varpi \left( -\left( A^2+B^2\right) \right) }-A \sqrt{-\varpi } \cosh \left( \frac{2 \sqrt{-\varpi } \left( \varrho g+2 \sqrt{2 \varpi -\omega } \left( \frac{1}{\Gamma (\varrho )}+t\right) ^{\varrho }+\left( \frac{1}{\Gamma (\varrho )}+x\right) ^{\varrho }\right) }{\varrho }\right) }. \end{aligned}$$

68 $$\begin{aligned} & \Upsilon _{-1}(x, t)_{1,1}=\frac{\sqrt{\varpi ^2-g_1^2} \exp \left( i \left( \frac{\omega \left( \frac{1}{\Gamma (\varrho )}+t\right) ^{\varrho }}{\varrho }-\frac{\sqrt{2 \varpi -\omega } \left( \frac{1}{\Gamma (\varrho )}+x\right) ^{\varrho }}{\varrho }\right) \right) \left( A \sinh \left( \frac{2 \sqrt{-\varpi } \left( \varrho g+2 \sqrt{2 \varpi -\omega } \left( \frac{1}{\Gamma (\varrho )}+t\right) ^{\varrho }+\left( \frac{1}{\Gamma (\varrho )}+x\right) ^{\varrho }\right) }{\varrho }\right) +B\right) }{\sqrt{\varpi \left( -\left( A^2+B^2\right) \right) }-A \sqrt{-\varpi } \cosh \left( \frac{2 \sqrt{-\varpi } \left( \varrho g+2 \sqrt{2 \varpi -\omega } \left( \frac{1}{\Gamma (\varrho )}+t\right) ^{\varrho }+\left( \frac{1}{\Gamma (\varrho )}+x\right) ^{\varrho }\right) }{\varrho }\right) }. \end{aligned}$$

69 $$\begin{aligned} & \Upsilon _{+1}(x, t)_{2,1}=\frac{\sqrt{\varpi ^2-g_1^2} \exp \left( i \left( \frac{\omega \left( \frac{1}{\Gamma (\varrho )}+t\right) ^{\varrho }}{\varrho }-\frac{\sqrt{2 \varpi -\omega } \left( \frac{1}{\Gamma (\varrho )}+x\right) ^{\varrho }}{\varrho }\right) \right) \left( A \sinh \left( \frac{2 \sqrt{-\varpi } \left( \varrho g+2 \sqrt{2 \varpi -\omega } \left( \frac{1}{\Gamma (\varrho )}+t\right) ^{\varrho }+\left( \frac{1}{\Gamma (\varrho )}+x\right) ^{\varrho }\right) }{\varrho }\right) +B\right) }{\sqrt{\varpi \left( -\left( A^2+B^2\right) \right) }-A \sqrt{-\varpi } \cosh \left( \frac{2 \sqrt{-\varpi } \left( \varrho g+2 \sqrt{2 \varpi -\omega } \left( \frac{1}{\Gamma (\varrho )}+t\right) ^{\varrho }+\left( \frac{1}{\Gamma (\varrho )}+x\right) ^{\varrho }\right) }{\varrho }\right) }. \end{aligned}$$

70 $$\begin{aligned} & \Upsilon _{0}(x, t)_{2,1}=-\frac{g_1 \exp \left( i \left( \frac{\omega \left( \frac{1}{\Gamma (\varrho )}+t\right) ^{\varrho }}{\varrho }-\frac{\sqrt{2 \varpi -\omega } \left( \frac{1}{\Gamma (\varrho )}+x\right) ^{\varrho }}{\varrho }\right) \right) \left( A \sinh \left( \frac{2 \sqrt{-\varpi } \left( \varrho g+2 \sqrt{2 \varpi -\omega } \left( \frac{1}{\Gamma (\varrho )}+t\right) ^{\varrho }+\left( \frac{1}{\Gamma (\varrho )}+x\right) ^{\varrho }\right) }{\varrho }\right) +B\right) }{\sqrt{\varpi \left( -\left( A^2+B^2\right) \right) }-A \sqrt{-\varpi } \cosh \left( \frac{2 \sqrt{-\varpi } \left( \varrho g+2 \sqrt{2 \varpi -\omega } \left( \frac{1}{\Gamma (\varrho )}+t\right) ^{\varrho }+\left( \frac{1}{\Gamma (\varrho )}+x\right) ^{\varrho }\right) }{\varrho }\right) }. \end{aligned}$$

71 $$\begin{aligned} & \Upsilon _{-1}(x, t)_{2,1}=-\frac{\sqrt{\varpi ^2-g_1^2} \exp \left( i \left( \frac{\omega \left( \frac{1}{\Gamma (\varrho )}+t\right) ^{\varrho }}{\varrho }-\frac{\sqrt{2 \varpi -\omega } \left( \frac{1}{\Gamma (\varrho )}+x\right) ^{\varrho }}{\varrho }\right) \right) \left( A \sinh \left( \frac{2 \sqrt{-\varpi } \left( \varrho g+2 \sqrt{2 \varpi -\omega } \left( \frac{1}{\Gamma (\varrho )}+t\right) ^{\varrho }+\left( \frac{1}{\Gamma (\varrho )}+x\right) ^{\varrho }\right) }{\varrho }\right) +B\right) }{\sqrt{\varpi \left( -\left( A^2+B^2\right) \right) }-A \sqrt{-\varpi } \cosh \left( \frac{2 \sqrt{-\varpi } \left( \varrho g+2 \sqrt{2 \varpi -\omega } \left( \frac{1}{\Gamma (\varrho )}+t\right) ^{\varrho }+\left( \frac{1}{\Gamma (\varrho )}+x\right) ^{\varrho }\right) }{\varrho }\right) }. \end{aligned}$$

72 $$\begin{aligned} & \Upsilon _{+1}(x, t)_{3,1}=\frac{\sqrt{-\varpi } \sqrt{\varpi ^2-g_1^2} \exp \left( i \left( \frac{\omega \left( \frac{1}{\Gamma (\varrho )}+t\right) ^{\varrho }}{\varrho }-\frac{\sqrt{2 \varpi -\omega } \left( \frac{1}{\Gamma (\varrho )}+x\right) ^{\varrho }}{\varrho }\right) \right) }{\varpi -\frac{2 A \varpi }{A+\cosh \left( \frac{2 \sqrt{-\varpi } \left( \varrho g+2 \sqrt{2 \varpi -\omega } \left( \frac{1}{\Gamma (\varrho )}+t\right) ^{\varrho }+\left( \frac{1}{\Gamma (\varrho )}+x\right) ^{\varrho }\right) }{\varrho }\right) -\sinh \left( \frac{2 \sqrt{-\varpi } \left( \varrho g+2 \sqrt{2 \varpi -\omega } \left( \frac{1}{\Gamma (\varrho )}+t\right) ^{\varrho }+\left( \frac{1}{\Gamma (\varrho )}+x\right) ^{\varrho }\right) }{\varrho }\right) }}. \end{aligned}$$

73 $$\begin{aligned} & \Upsilon _{0}(x, t)_{3,1}=\frac{g_1 \exp \left( i \left( \frac{\omega \left( \frac{1}{\Gamma (\varrho )}+t\right) ^{\varrho }}{\varrho }-\frac{\sqrt{2 \varpi -\omega } \left( \frac{1}{\Gamma (\varrho )}+x\right) ^{\varrho }}{\varrho }\right) \right) }{\sqrt{-\varpi } \left( 1-\frac{2 A}{A+\cosh \left( \frac{2 \sqrt{-\varpi } \left( \varrho g+2 \sqrt{2 \varpi -\omega } \left( \frac{1}{\Gamma (\varrho )}+t\right) ^{\varrho }+\left( \frac{1}{\Gamma (\varrho )}+x\right) ^{\varrho }\right) }{\varrho }\right) -\sinh \left( \frac{2 \sqrt{-\varpi } \left( \varrho g+2 \sqrt{2 \varpi -\omega } \left( \frac{1}{\Gamma (\varrho )}+t\right) ^{\varrho }+\left( \frac{1}{\Gamma (\varrho )}+x\right) ^{\varrho }\right) }{\varrho }\right) }\right) }. \end{aligned}$$

74 $$\begin{aligned} & \Upsilon _{-1}(x, t)_{3,1}=\frac{\sqrt{\varpi ^2-g_1^2} \exp \left( i \left( \frac{\omega \left( \frac{1}{\Gamma (\varrho )}+t\right) ^{\varrho }}{\varrho }-\frac{\sqrt{2 \varpi -\omega } \left( \frac{1}{\Gamma (\varrho )}+x\right) ^{\varrho }}{\varrho }\right) \right) }{\sqrt{-\varpi } \left( 1-\frac{2 A}{A+\cosh \left( \frac{2 \sqrt{-\varpi } \left( \varrho g+2 \sqrt{2 \varpi -\omega } \left( \frac{1}{\Gamma (\varrho )}+t\right) ^{\varrho }+\left( \frac{1}{\Gamma (\varrho )}+x\right) ^{\varrho }\right) }{\varrho }\right) -\sinh \left( \frac{2 \sqrt{-\varpi } \left( \varrho g+2 \sqrt{2 \varpi -\omega } \left( \frac{1}{\Gamma (\varrho )}+t\right) ^{\varrho }+\left( \frac{1}{\Gamma (\varrho )}+x\right) ^{\varrho }\right) }{\varrho }\right) }\right) }. \end{aligned}$$

75 $$\begin{aligned} & \Upsilon _{+1}(x, t)_{4,1}=\frac{\varpi \sqrt{\varpi ^2-g_1^2} \exp \left( i \left( \frac{\omega \left( \frac{1}{\Gamma (\varrho )}+t\right) ^{\varrho }}{\varrho }-\frac{\sqrt{2 \varpi -\omega } \left( \frac{1}{\Gamma (\varrho )}+x\right) ^{\varrho }}{\varrho }\right) \right) }{(-\varpi )^{3/2} \left( \frac{2 A}{A+\cosh \left( \frac{2 \sqrt{-\varpi } \left( \varrho g+2 \sqrt{2 \varpi -\omega } \left( \frac{1}{\Gamma (\varrho )}+t\right) ^{\varrho }+\left( \frac{1}{\Gamma (\varrho )}+x\right) ^{\varrho }\right) }{\varrho }\right) -\sinh \left( \frac{2 \sqrt{-\varpi } \left( \varrho g+2 \sqrt{2 \varpi -\omega } \left( \frac{1}{\Gamma (\varrho )}+t\right) ^{\varrho }+\left( \frac{1}{\Gamma (\varrho )}+x\right) ^{\varrho }\right) }{\varrho }\right) }-1\right) }. \end{aligned}$$

76 $$\begin{aligned} & \Upsilon _{0}(x, t)_{4,1}=\frac{g_1 \exp \left( i \left( \frac{\omega \left( \frac{1}{\Gamma (\varrho )}+t\right) ^{\varrho }}{\varrho }-\frac{\sqrt{2 \varpi -\omega } \left( \frac{1}{\Gamma (\varrho )}+x\right) ^{\varrho }}{\varrho }\right) \right) }{\sqrt{-\varpi } \left( \frac{2 A}{A+\cosh \left( \frac{2 \sqrt{-\varpi } \left( \varrho g+2 \sqrt{2 \varpi -\omega } \left( \frac{1}{\Gamma (\varrho )}+t\right) ^{\varrho }+\left( \frac{1}{\Gamma (\varrho )}+x\right) ^{\varrho }\right) }{\varrho }\right) -\sinh \left( \frac{2 \sqrt{-\varpi } \left( \varrho g+2 \sqrt{2 \varpi -\omega } \left( \frac{1}{\Gamma (\varrho )}+t\right) ^{\varrho }+\left( \frac{1}{\Gamma (\varrho )}+x\right) ^{\varrho }\right) }{\varrho }\right) }-1\right) }. \end{aligned}$$

77 $$\begin{aligned} & \Upsilon _{-1}(x, t)_{4,1}=\frac{\sqrt{\varpi ^2-g_1^2} \exp \left( i \left( \frac{\omega \left( \frac{1}{\Gamma (\varrho )}+t\right) ^{\varrho }}{\varrho }-\frac{\sqrt{2 \varpi -\omega } \left( \frac{1}{\Gamma (\varrho )}+x\right) ^{\varrho }}{\varrho }\right) \right) }{\sqrt{-\varpi } \left( \frac{2 A}{A+\cosh \left( \frac{2 \sqrt{-\varpi } \left( \varrho g+2 \sqrt{2 \varpi -\omega } \left( \frac{1}{\Gamma (\varrho )}+t\right) ^{\varrho }+\left( \frac{1}{\Gamma (\varrho )}+x\right) ^{\varrho }\right) }{\varrho }\right) -\sinh \left( \frac{2 \sqrt{-\varpi } \left( \varrho g+2 \sqrt{2 \varpi -\omega } \left( \frac{1}{\Gamma (\varrho )}+t\right) ^{\varrho }+\left( \frac{1}{\Gamma (\varrho )}+x\right) ^{\varrho }\right) }{\varrho }\right) }-1\right) }. \end{aligned}$$

** Case-2** **Trigonometric solution**:

If $$\varpi >0$$,

78 $$\begin{aligned} & \Upsilon _{+1}(x, t)_{1,2}=\frac{\sqrt{\varpi ^2-g_1^2} \exp \left( i \left( \frac{\omega \left( \frac{1}{\Gamma (\varrho )}+t\right) ^{\varrho }}{\varrho }-\frac{\sqrt{2 \varpi -\omega } \left( \frac{1}{\Gamma (\varrho )}+x\right) ^{\varrho }}{\varrho }\right) \right) \left( A \sin \left( \frac{2 \sqrt{\varpi } \left( \varrho g+2 \sqrt{2 \varpi -\omega } \left( \frac{1}{\Gamma (\varrho )}+t\right) ^{\varrho }+\left( \frac{1}{\Gamma (\varrho )}+x\right) ^{\varrho }\right) }{\varrho }\right) +B\right) }{A \sqrt{\varpi } \cos \left( \frac{2 \sqrt{\varpi } \left( \varrho g+2 \sqrt{2 \varpi -\omega } \left( \frac{1}{\Gamma (\varrho )}+t\right) ^{\varrho }+\left( \frac{1}{\Gamma (\varrho )}+x\right) ^{\varrho }\right) }{\varrho }\right) -\sqrt{\varpi \left( A^2-B^2\right) }}. \end{aligned}$$

79 $$\begin{aligned} & \Upsilon _{0}(x, t)_{1,2}=\frac{g_1 \exp \left( i \left( \frac{\omega \left( \frac{1}{\Gamma (\varrho )}+t\right) ^{\varrho }}{\varrho }-\frac{\sqrt{2 \varpi -\omega } \left( \frac{1}{\Gamma (\varrho )}+x\right) ^{\varrho }}{\varrho }\right) \right) \left( A \sin \left( \frac{2 \sqrt{\varpi } \left( \varrho g+2 \sqrt{2 \varpi -\omega } \left( \frac{1}{\Gamma (\varrho )}+t\right) ^{\varrho }+\left( \frac{1}{\Gamma (\varrho )}+x\right) ^{\varrho }\right) }{\varrho }\right) +B\right) }{\sqrt{\varpi \left( A^2-B^2\right) }-A \sqrt{\varpi } \cos \left( \frac{2 \sqrt{\varpi } \left( \varrho g+2 \sqrt{2 \varpi -\omega } \left( \frac{1}{\Gamma (\varrho )}+t\right) ^{\varrho }+\left( \frac{1}{\Gamma (\varrho )}+x\right) ^{\varrho }\right) }{\varrho }\right) }. \end{aligned}$$

80 $$\begin{aligned} & \Upsilon _{-1}(x, t)_{1,2}=-\frac{\sqrt{\varpi ^2-g_1^2} \exp \left( i \left( \frac{\omega \left( \frac{1}{\Gamma (\varrho )}+t\right) ^{\varrho }}{\varrho }-\frac{\sqrt{2 \varpi -\omega } \left( \frac{1}{\Gamma (\varrho )}+x\right) ^{\varrho }}{\varrho }\right) \right) \left( A \sin \left( \frac{2 \sqrt{\varpi } \left( \varrho g+2 \sqrt{2 \varpi -\omega } \left( \frac{1}{\Gamma (\varrho )}+t\right) ^{\varrho }+\left( \frac{1}{\Gamma (\varrho )}+x\right) ^{\varrho }\right) }{\varrho }\right) +B\right) }{A \sqrt{\varpi } \cos \left( \frac{2 \sqrt{\varpi } \left( \varrho g+2 \sqrt{2 \varpi -\omega } \left( \frac{1}{\Gamma (\varrho )}+t\right) ^{\varrho }+\left( \frac{1}{\Gamma (\varrho )}+x\right) ^{\varrho }\right) }{\varrho }\right) -\sqrt{\varpi \left( A^2-B^2\right) }}. \end{aligned}$$

81 $$\begin{aligned} & \Upsilon _{+1}(x, t)_{2,2}=-\frac{\sqrt{\varpi ^2-g_1^2} \exp \left( i \left( \frac{\omega \left( \frac{1}{\Gamma (\varrho )}+t\right) ^{\varrho }}{\varrho }-\frac{\sqrt{2 \varpi -\omega } \left( \frac{1}{\Gamma (\varrho )}+x\right) ^{\varrho }}{\varrho }\right) \right) \left( A \sin \left( \frac{2 \sqrt{\varpi } \left( \varrho g+2 \sqrt{2 \varpi -\omega } \left( \frac{1}{\Gamma (\varrho )}+t\right) ^{\varrho }+\left( \frac{1}{\Gamma (\varrho )}+x\right) ^{\varrho }\right) }{\varrho }\right) +B\right) }{A \sqrt{\varpi } \cos \left( \frac{2 \sqrt{\varpi } \left( \varrho g+2 \sqrt{2 \varpi -\omega } \left( \frac{1}{\Gamma (\varrho )}+t\right) ^{\varrho }+\left( \frac{1}{\Gamma (\varrho )}+x\right) ^{\varrho }\right) }{\varrho }\right) -\sqrt{\varpi \left( A^2-B^2\right) }}. \end{aligned}$$

82 $$\begin{aligned} & \Upsilon _{0}(x, t)_{2,2}=-\frac{g_1 \exp \left( i \left( \frac{\omega \left( \frac{1}{\Gamma (\varrho )}+t\right) ^{\varrho }}{\varrho }-\frac{\sqrt{2 \varpi -\omega } \left( \frac{1}{\Gamma (\varrho )}+x\right) ^{\varrho }}{\varrho }\right) \right) \left( A \sin \left( \frac{2 \sqrt{\varpi } \left( \varrho g+2 \sqrt{2 \varpi -\omega } \left( \frac{1}{\Gamma (\varrho )}+t\right) ^{\varrho }+\left( \frac{1}{\Gamma (\varrho )}+x\right) ^{\varrho }\right) }{\varrho }\right) +B\right) }{\sqrt{\varpi \left( A^2-B^2\right) }-A \sqrt{\varpi } \cos \left( \frac{2 \sqrt{\varpi } \left( \varrho g+2 \sqrt{2 \varpi -\omega } \left( \frac{1}{\Gamma (\varrho )}+t\right) ^{\varrho }+\left( \frac{1}{\Gamma (\varrho )}+x\right) ^{\varrho }\right) }{\varrho }\right) }. \end{aligned}$$

83 $$\begin{aligned} & \Upsilon _{-1}(x, t)_{2,2}=\frac{\sqrt{\varpi ^2-g_1^2} \exp \left( i \left( \frac{\omega \left( \frac{1}{\Gamma (\varrho )}+t\right) ^{\varrho }}{\varrho }-\frac{\sqrt{2 \varpi -\omega } \left( \frac{1}{\Gamma (\varrho )}+x\right) ^{\varrho }}{\varrho }\right) \right) \left( A \sin \left( \frac{2 \sqrt{\varpi } \left( \varrho g+2 \sqrt{2 \varpi -\omega } \left( \frac{1}{\Gamma (\varrho )}+t\right) ^{\varrho }+\left( \frac{1}{\Gamma (\varrho )}+x\right) ^{\varrho }\right) }{\varrho }\right) +B\right) }{A \sqrt{\varpi } \cos \left( \frac{2 \sqrt{\varpi } \left( \varrho g+2 \sqrt{2 \varpi -\omega } \left( \frac{1}{\Gamma (\varrho )}+t\right) ^{\varrho }+\left( \frac{1}{\Gamma (\varrho )}+x\right) ^{\varrho }\right) }{\varrho }\right) -\sqrt{\varpi \left( A^2-B^2\right) }}. \end{aligned}$$

84 $$\begin{aligned} & \Upsilon _{+1}(x, t)_{3,2}=\frac{i \sqrt{\varpi ^2-g_1^2} \exp \left( i \left( \frac{\omega \left( \frac{1}{\Gamma (\varrho )}+t\right) ^{\varrho }}{\varrho }-\frac{\sqrt{2 \varpi -\omega } \left( \frac{1}{\Gamma (\varrho )}+x\right) ^{\varrho }}{\varrho }\right) \right) }{\sqrt{\varpi } \left( 1-\frac{2 A}{A+\cos \left( \frac{2 \sqrt{\varpi } \left( \varrho g+2 \sqrt{2 \varpi -\omega } \left( \frac{1}{\Gamma (\varrho )}+t\right) ^{\varrho }+\left( \frac{1}{\Gamma (\varrho )}+x\right) ^{\varrho }\right) }{\varrho }\right) -i \sin \left( \frac{2 \sqrt{\varpi } \left( \varrho g+2 \sqrt{2 \varpi -\omega } \left( \frac{1}{\Gamma (\varrho )}+t\right) ^{\varrho }+\left( \frac{1}{\Gamma (\varrho )}+x\right) ^{\varrho }\right) }{\varrho }\right) }\right) }. \end{aligned}$$

85 $$\begin{aligned} & \Upsilon _{0}(x, t)_{3,2}=-\frac{i g_1 \exp \left( i \left( \frac{\omega \left( \frac{1}{\Gamma (\varrho )}+t\right) ^{\varrho }}{\varrho }-\frac{\sqrt{2 \varpi -\omega } \left( \frac{1}{\Gamma (\varrho )}+x\right) ^{\varrho }}{\varrho }\right) \right) }{\sqrt{\varpi } \left( 1-\frac{2 A}{A+\cos \left( \frac{2 \sqrt{\varpi } \left( \varrho g+2 \sqrt{2 \varpi -\omega } \left( \frac{1}{\Gamma (\varrho )}+t\right) ^{\varrho }+\left( \frac{1}{\Gamma (\varrho )}+x\right) ^{\varrho }\right) }{\varrho }\right) -i \sin \left( \frac{2 \sqrt{\varpi } \left( \varrho g+2 \sqrt{2 \varpi -\omega } \left( \frac{1}{\Gamma (\varrho )}+t\right) ^{\varrho }+\left( \frac{1}{\Gamma (\varrho )}+x\right) ^{\varrho }\right) }{\varrho }\right) }\right) }. \end{aligned}$$

86 $$\begin{aligned} & \Upsilon _{-1}(x, t)_{3,2}=-\frac{i \sqrt{\varpi ^2-g_1^2} \exp \left( i \left( \frac{\omega \left( \frac{1}{\Gamma (\varrho )}+t\right) ^{\varrho }}{\varrho }-\frac{\sqrt{2 \varpi -\omega } \left( \frac{1}{\Gamma (\varrho )}+x\right) ^{\varrho }}{\varrho }\right) \right) }{\sqrt{\varpi } \left( 1-\frac{2 A}{A+\cos \left( \frac{2 \sqrt{\varpi } \left( \varrho g+2 \sqrt{2 \varpi -\omega } \left( \frac{1}{\Gamma (\varrho )}+t\right) ^{\varrho }+\left( \frac{1}{\Gamma (\varrho )}+x\right) ^{\varrho }\right) }{\varrho }\right) -i \sin \left( \frac{2 \sqrt{\varpi } \left( \varrho g+2 \sqrt{2 \varpi -\omega } \left( \frac{1}{\Gamma (\varrho )}+t\right) ^{\varrho }+\left( \frac{1}{\Gamma (\varrho )}+x\right) ^{\varrho }\right) }{\varrho }\right) }\right) }. \end{aligned}$$

87 $$\begin{aligned} & \Upsilon _{+1}(x, t)_{4,2}=-\frac{i \sqrt{\varpi ^2-g_1^2} \exp \left( i \left( \frac{\omega \left( \frac{1}{\Gamma (\varrho )}+t\right) ^{\varrho }}{\varrho }-\frac{\sqrt{2 \varpi -\omega } \left( \frac{1}{\Gamma (\varrho )}+x\right) ^{\varrho }}{\varrho }\right) \right) }{\sqrt{\varpi } \left( 1-\frac{2 A}{A+\cos \left( \frac{2 \sqrt{\varpi } \left( \varrho g+2 \sqrt{2 \varpi -\omega } \left( \frac{1}{\Gamma (\varrho )}+t\right) ^{\varrho }+\left( \frac{1}{\Gamma (\varrho )}+x\right) ^{\varrho }\right) }{\varrho }\right) +i \sin \left( \frac{2 \sqrt{\varpi } \left( \varrho g+2 \sqrt{2 \varpi -\omega } \left( \frac{1}{\Gamma (\varrho )}+t\right) ^{\varrho }+\left( \frac{1}{\Gamma (\varrho )}+x\right) ^{\varrho }\right) }{\varrho }\right) }\right) }. \end{aligned}$$

88 $$\begin{aligned} & \Upsilon _{0}(x, t)_{4,2}=\frac{i g_1 \exp \left( i \left( \frac{\omega \left( \frac{1}{\Gamma (\varrho )}+t\right) ^{\varrho }}{\varrho }-\frac{\sqrt{2 \varpi -\omega } \left( \frac{1}{\Gamma (\varrho )}+x\right) ^{\varrho }}{\varrho }\right) \right) }{\sqrt{\varpi } \left( 1-\frac{2 A}{A+\cos \left( \frac{2 \sqrt{\varpi } \left( \varrho g+2 \sqrt{2 \varpi -\omega } \left( \frac{1}{\Gamma (\varrho )}+t\right) ^{\varrho }+\left( \frac{1}{\Gamma (\varrho )}+x\right) ^{\varrho }\right) }{\varrho }\right) +i \sin \left( \frac{2 \sqrt{\varpi } \left( \varrho g+2 \sqrt{2 \varpi -\omega } \left( \frac{1}{\Gamma (\varrho )}+t\right) ^{\varrho }+\left( \frac{1}{\Gamma (\varrho )}+x\right) ^{\varrho }\right) }{\varrho }\right) }\right) }. \end{aligned}$$

89 $$\begin{aligned} & \Upsilon _{-1}(x, t)_{4,2}=\frac{i \sqrt{\varpi ^2-g_1^2} \exp \left( i \left( \frac{\omega \left( \frac{1}{\Gamma (\varrho )}+t\right) ^{\varrho }}{\varrho }-\frac{\sqrt{2 \varpi -\omega } \left( \frac{1}{\Gamma (\varrho )}+x\right) ^{\varrho }}{\varrho }\right) \right) }{\sqrt{\varpi } \left( 1-\frac{2 A}{A+\cos \left( \frac{2 \sqrt{\varpi } \left( \varrho g+2 \sqrt{2 \varpi -\omega } \left( \frac{1}{\Gamma (\varrho )}+t\right) ^{\varrho }+\left( \frac{1}{\Gamma (\varrho )}+x\right) ^{\varrho }\right) }{\varrho }\right) +i \sin \left( \frac{2 \sqrt{\varpi } \left( \varrho g+2 \sqrt{2 \varpi -\omega } \left( \frac{1}{\Gamma (\varrho )}+t\right) ^{\varrho }+\left( \frac{1}{\Gamma (\varrho )}+x\right) ^{\varrho }\right) }{\varrho }\right) }\right) }. \end{aligned}$$

** Case-3** **Plane wave solution**:

If $$\varpi =0$$,

90 $$\begin{aligned} & \Upsilon _{+1}(x, t)_{1,3}=\frac{\sqrt{-g_1^2} \exp \left( i \left( \frac{\omega \left( \frac{1}{\Gamma (\varrho )}+t\right) ^{\varrho }}{\varrho }-\frac{\sqrt{-\omega } \left( \frac{1}{\Gamma (\varrho )}+x\right) ^{\varrho }}{\varrho }\right) \right) \left( \varrho g+2 \sqrt{-\omega } \left( \frac{1}{\Gamma (\varrho )}+t\right) ^{\varrho }+\left( \frac{1}{\Gamma (\varrho )}+x\right) ^{\varrho }\right) }{\varrho }. \end{aligned}$$

91 $$\begin{aligned} & \Upsilon _{0}(x, t)_{1,3}=-\frac{g_1 \exp \left( i \left( \frac{\omega \left( \frac{1}{\Gamma (\varrho )}+t\right) ^{\varrho }}{\varrho }-\frac{\sqrt{-\omega } \left( \frac{1}{\Gamma (\varrho )}+x\right) ^{\varrho }}{\varrho }\right) \right) \left( \varrho g+2 \sqrt{-\omega } \left( \frac{1}{\Gamma (\varrho )}+t\right) ^{\varrho }+\left( \frac{1}{\Gamma (\varrho )}+x\right) ^{\varrho }\right) }{\varrho }. \end{aligned}$$

92 $$\begin{aligned} & \Upsilon _{-1}(x, t)_{1,3}=-\frac{\sqrt{-g_1^2} \exp \left( i \left( \frac{\omega \left( \frac{1}{\Gamma (\varrho )}+t\right) ^{\varrho }}{\varrho }-\frac{\sqrt{-\omega } \left( \frac{1}{\Gamma (\varrho )}+x\right) ^{\varrho }}{\varrho }\right) \right) \left( \varrho g+2 \sqrt{-\omega } \left( \frac{1}{\Gamma (\varrho )}+t\right) ^{\varrho }+\left( \frac{1}{\Gamma (\varrho )}+x\right) ^{\varrho }\right) }{\varrho }. \end{aligned}$$

**Remark**. Similarly, solutions for Set-2 and Set-3 can be received.

## Results and discussion

**Fig. 1:**
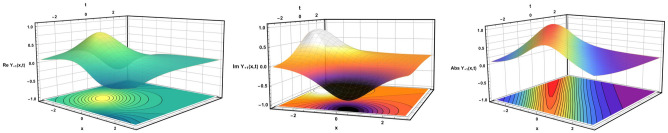
The bright wave structure to Eq. (33) exhibits a localized peak intensity on a continuous background in 3D and contour plots, adjusted by free parameters: $\varrho =.98,~ a_1=0.85,~k_0=0.05,~\mu =-0.75,~\tau =0.7,~\text{ and }~\omega =0.55.$.

**Fig. 2:**
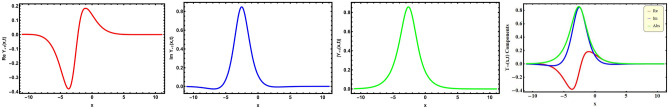
The 2D graphs of Eqs. (33, 34, 35) exemplify the absolute, imaginary, and real parts, and their combined behavior at $t=1$.

**Fig. 3:**
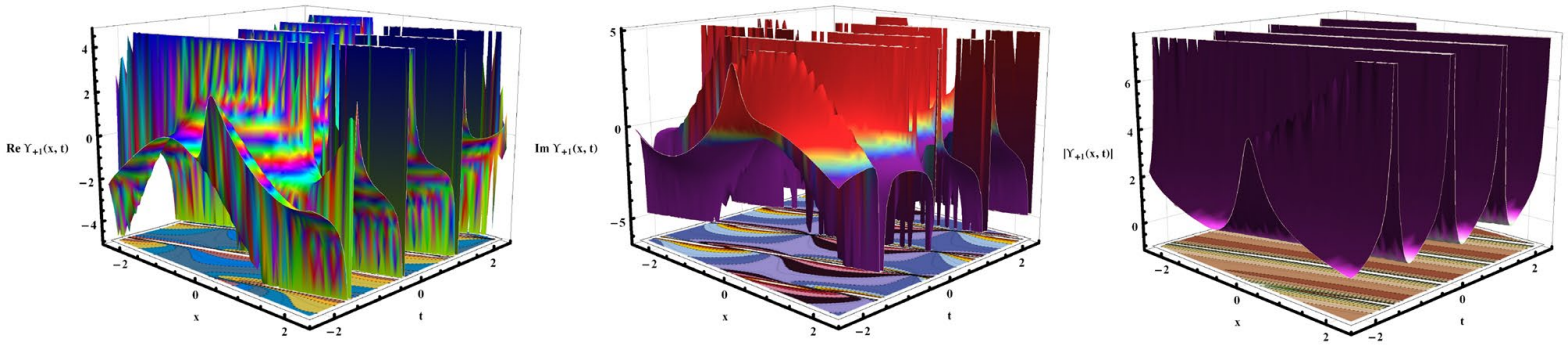
The soliton solution for Eqs. (48, 49, 50) denotes periodic singular solitons, exemplified by a repetitive pattern. To visualize this solution effectively, we choose specific values for the arbitrary free constants, yielding informative 3D and contour plots for $\varrho =.99,~ b_1=0.85,~k_0=0.04,~\mu =0.90,~\tau =1.1,~\text{ and }~\omega =0.5.$.

**Fig. 4:**
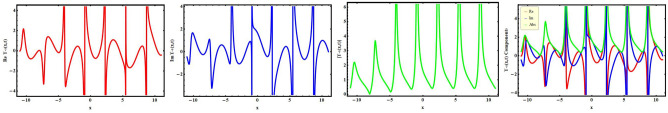
The 2D graphs of Eq. (48, 49, 50) exemplify the absolute, imaginary, and real parts, and their combined behavior at $t=1$.

**Fig. 5:**
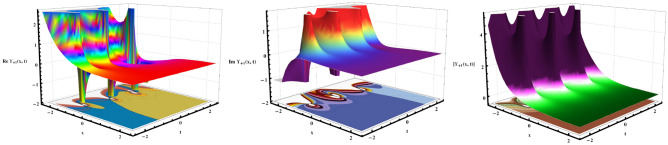
The dark soliton solution to Eq. (54, 55, 56) displays a localized intensity dip on a continuous wave background, as depicted in 3D and contour plots with suitable free parameters: $\varrho =.98,~ a_1=0.85,~k_0=0.05,~\mu =-0.75,~\tau =0.7,~\text{ and }~\omega =0.55.$.

**Fig. 6:**
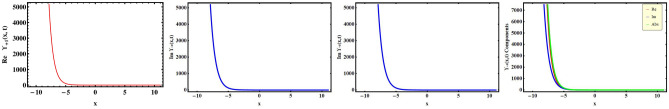
The 2D graphs of Eq. (54, 55, 56) exemplify the absolute, imaginary, and real parts, and their combined behavior at $t=1$.

**Fig. 7:**
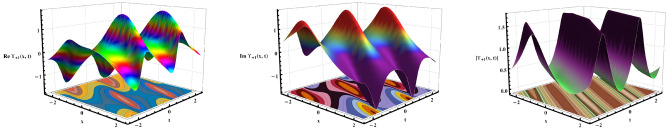
The Eq. (66, 67, 68) visually shows hyperbolic behavior, with its arbitrary constants affecting the formation of solitons in 3D and contour plots for $g_1=0.07,~A=-1.6,~\varrho =0.98,~B=1.5,~g=0.2,~\omega =0.17,~\text{ and }~\varpi =0.5.$.

**Fig. 8:**
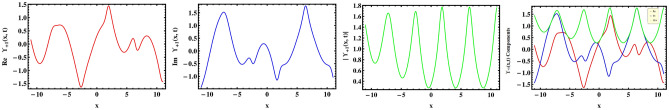
The 2D graphs of Eq. (66, 67, 68) exemplify the absolute, imaginary, and real parts, and their combined behavior at $t=1$.

This study exhibits a comprehensive analysis of exact soliton solutions attained through the aforementioned approaches, expressed as plane wave, hyperbolic, and trigonometric functions, with their dynamics explicated through advanced 3D surface plots, 2D profiles, and contour maps (Figures 1, 2, 3, 4, 5, 6, 7 and 8). By means of graphical analysis we expose the underlying mechanisms of nonlinear wave phenomena for the $\beta$-fractional derivative model which shows that the soliton is more stable and the wave profile is smoother as the fractional order increases which makes it more suitable to describe optical solitons in three-component GP systems. The 2D plots (Figures 2, 3, 4, 5, 6, 7 and 8) clearly illustrate the features of the solution via real, imaginary, absolute and composite plots which ensures the reliability of our method in various applications in optical fiber communications, plasma physics, fluid dynamics and mathematical biology. These computationally efficient techniques not only supply engineers with useful approaches to build models for photonic devices but also disclose rich soliton structures with direct applications to nonlinear optical transmission systems, quantum fluid dynamics and wave propagation in dispersive media. The results confirm our methodological framework. In addition, the $\beta$-derivative reveals promising potential to promote optical soliton research. Undoubtedly, the remarkable adaptability and wide range of applications in various physical models will pave new approaches to investigate nonlinear phenomena in many other physical settings. In order to present the novelty of this work, we make a comparative analysis of our work with the published works. In 2021, the authors in^44^ studied the governing model and constructed some various soliton profiles by the extended sinh-Gordon equation expansion method. In 2023, the authors incite14g re-studied the governing model and obtained some new solutions by the direct algebraic method. In 2024, the authors in^46^ used two analytical techniques modified Sardar sub-equation method and enhanced modified extended tanh-expansion method to obtain some dispersive wave solutions. While in our research, we used the new EHFM and unified method to obtain some different wave structures and many of them are different with those in^44–46^, which shows the novelty of our research. We believe that the evaluated results are fresh and original and could be great addition in the theory of BEC and might be helpful for additional investigation of the GP Systems. Graphical simulations and a comparative analysis of some reported results is given below.

## Physical dynamics and applications of complex soliton wave patterns

Soliton solutions are stable, localized wave packets that maintain their shape and speed during propagation due to a balance between nonlinearity and dispersion. They are essential in nonlinear optics for preventing the light signal from attenuation during long distance optical fiber transmission and in BECs as coherent matter-wave structures that may be applied to explore quantum properties and superfluidity. The appearance of real and imaginary parts in soliton solutions enhances the understanding of wave behavior in complex-valued physical problems. Actually, in nonlinear models, respectively, in quantum mechanics, optical fiber, and fluid dynamics solutions appear of complex type, the imaginary part giving information about wave phase shift or intrinsic modes, to be observed in the imaginary part. However, the appearance of the real part is more obvious, but many important dynamical phenomena, like interference, stability properties, or energy interaction mechanisms, are actually revealed by the imaginary and the absolute value of the soliton, existing in purely real-valued solutions. Figures (1, 2, 3, 4, 5, 6, 7 and 8) illustrate this fact vividly by comparing the dynamic structure of the real part, the imaginary part, and the absolute form of the soliton. The practical impact of these solutions is immense. In optical fiber communications, they sharpen models of pulse propagation under dispersion and nonlinearity, thus optimizing high-speed data transmission. In plasma physics and fluid mechanics, the soliton analysis boosts the control over the interactions of waves and mitigates turbulence. Besides, in mathematical biology, such solutions may explain signal propagation in neurons or emerging shapes in biological tissues. The fractional configuration represents an extension of these applications because of the memory and the dissipation effects involved in the models, which are relevant for modeling different complex materials with hereditary effect. Thus, complex soliton solutions are important not only for a better theoretical understanding of the considered models but also for new technological advances.

## Conclusion

This study has extended new analytical approaches, namely EHFM and unified method with $\beta$-fractional derivative which can yield various exact solutions such as rational, hyperbolic, and trigonometric functions including various solitary wave shapes bright, dark, and singular solitons with different parameters for the complex tc GP equations. The effectiveness of the methods have been demonstrated by applying them in a systematic way to intricate NLPDEs. All obtained solutions have been mathematically verified using Mathematica through back substitution into the original equations. Two- and three-dimensional as well as contour plots of the wave solutions have been explained to reveal the fractional-order dynamical behavior. It has been shown that $\beta$-derivative produce smoother soliton evolution compared to the smoothness achieved by conventional techniques. Our computational analysis confirms the techniques superior accuracy and efficiency in handling nonlinear wave phenomena, while also enlightening an unexpectedly rich spectrum of soliton formations theoretically possible within the model. It has also shed light on an unexpectedly rich variety of soliton formations theoretically possible within the model. These have not only improved our current understanding of nonlinear dynamics in quantum systems but also provided a useful framework that can be extended to other NLPDE classes successfully by validating against benchmark problems. The novelty of our work has been established by a detailed comparison with the existing results reported in the literature. It has been shown that the obtained solutions are either new in functional forms, have wider range of parameters, or are more physically meaningful than the solutions reported before, which has improved our current understanding of the underlying nonlinear dynamics. Future research directions should focus on: (1) stochastic wave dynamics, (2) multiplicative noise effects, and (3) conducting systematic comparisons with established methods to enhance and validate the approaches robustness across distinct physical and mathematical contexts. The exhibited combination of analytical and computational accuracy have made these methods useful tools for both theoretical studies and practical engineering applications in nonlinear science.

## Data Availability

No datasets were generated or analysed during the current study.
